# Supplementary material for: ANKRD49 promotes the metastasis of NSCLC via activating JNK-ATF2/c-Jun-MMP-2/9 axis
Source: BMC Cancer. 2023 Nov 14;23:1108. doi: 10.1186/s12885-023-11612-9 (PMC10644579; doi:10.1186/s12885-023-11612-9)
Supplement: Supplementary file 1 — Additional file 1. Additional Figures [file 12885_2023_11612_MOESM1_ESM.docx]

**Supplementary Figures**

Fig. S1 Illustration the binding site of ATF2/c-Jun on MMP-2 or MMP-9 promoter region.

Fig. S2 ANKRD49 has no effect on cellular proliferation of H1299 cells.

Fig. S3 H1703 cells have the higher capability in cell migration and invasion than H1299 cells.

Fig. S4 ANKRD49 has no effect on cellular proliferation of H1703 cells.

Fig. S5 ANKRD49 promotes the lung metastasis of NSCLC cells in nude mice.

Fig. S6 Correlation analysis between H-score of ANKRD49, MMP-2, MMP-9, p-JNK, p-ATF2 or p-c-Jun and lung metastasis in mice injected with ANKRD49-OE H1299 cells.

Fig. S7 Correlation analysis between H-score of MMP-2, MMP-9, p-JNK, p-ATF2 or p-c-Jun and ANKRD49 in lung tissues of mice injected with ANKRD49-OE H1299 cells.

**
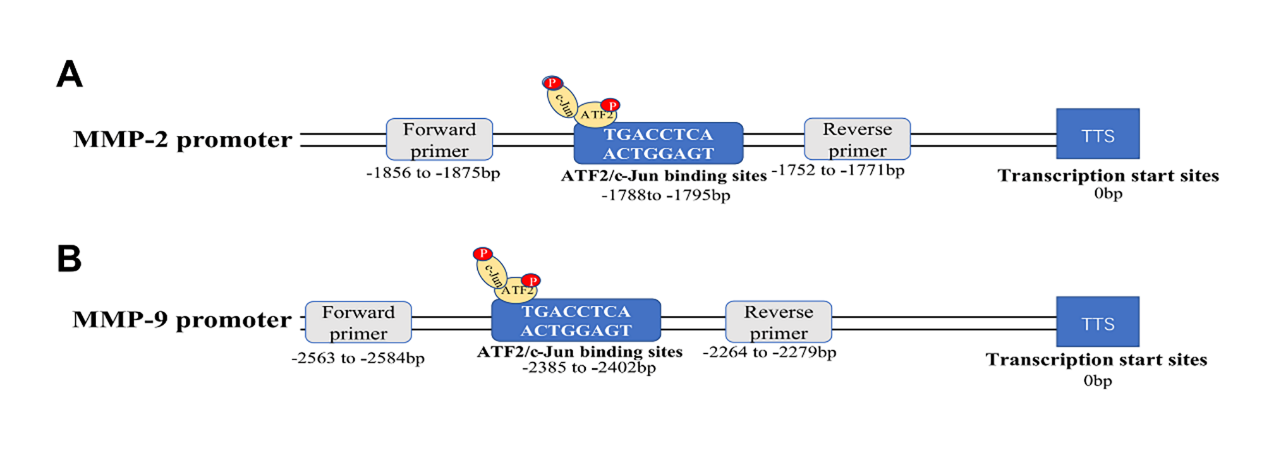
**

**Fig. S1 Illustration the binding site of ATF2/c-Jun on MMP-2 or MMP-9 promoter region.**

(A) illustration the binding site of ATF2/c-Jun on MMP-2 promoter region. (B) illustration the binding site of ATF2/c-Jun on MMP-9 promoter region.


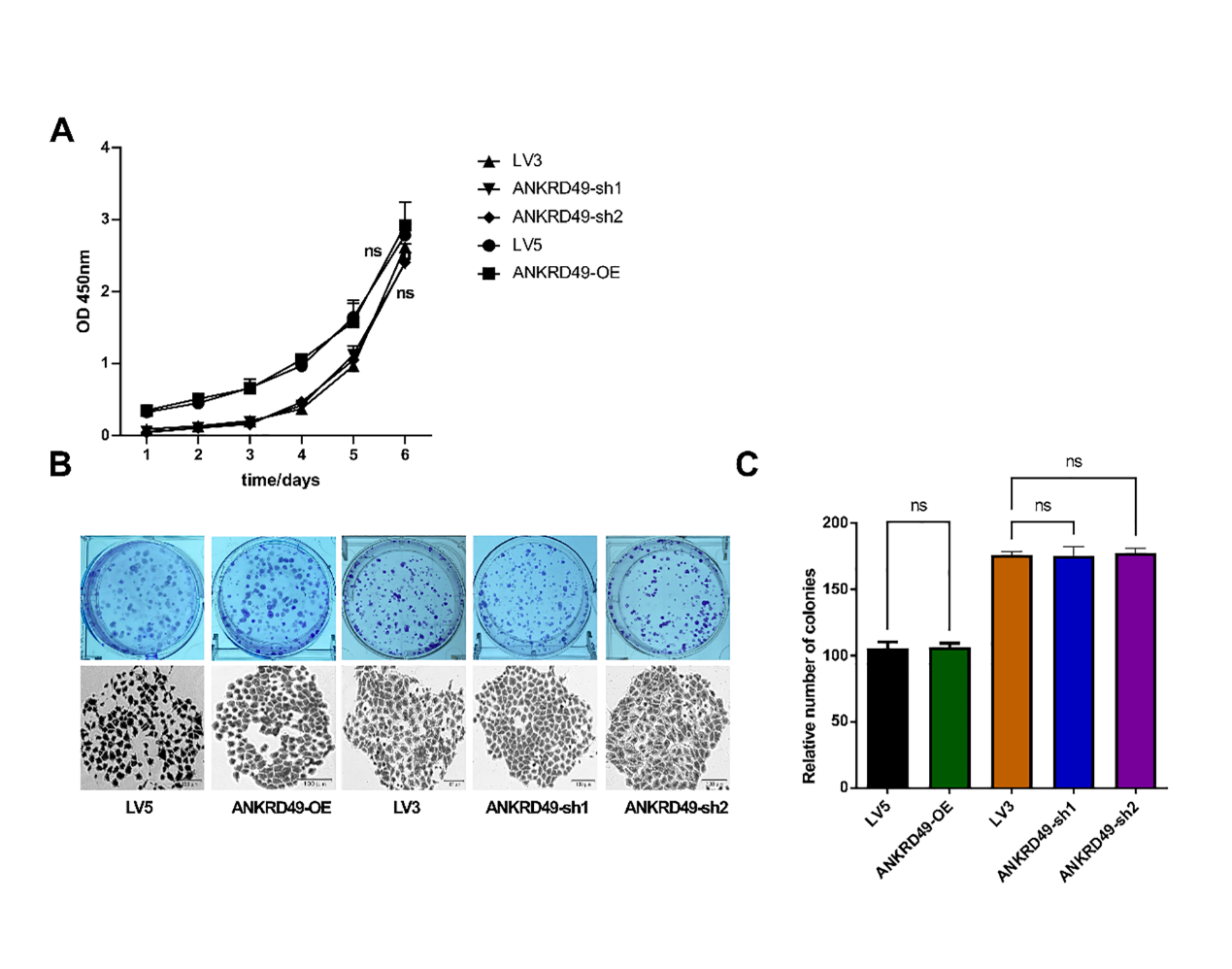


**Fig. S2 ANKRD49 has no effect on cellular proliferation of H1299 cells.**

Proliferation assays were performed using CCK8 (A) and colony formation (B, C) in ANKRD49-OE and ANKRD49-sh H1299 cells. All experiments were repeated independently three times. Data are presented as means ± standard deviation. ns, not significant.


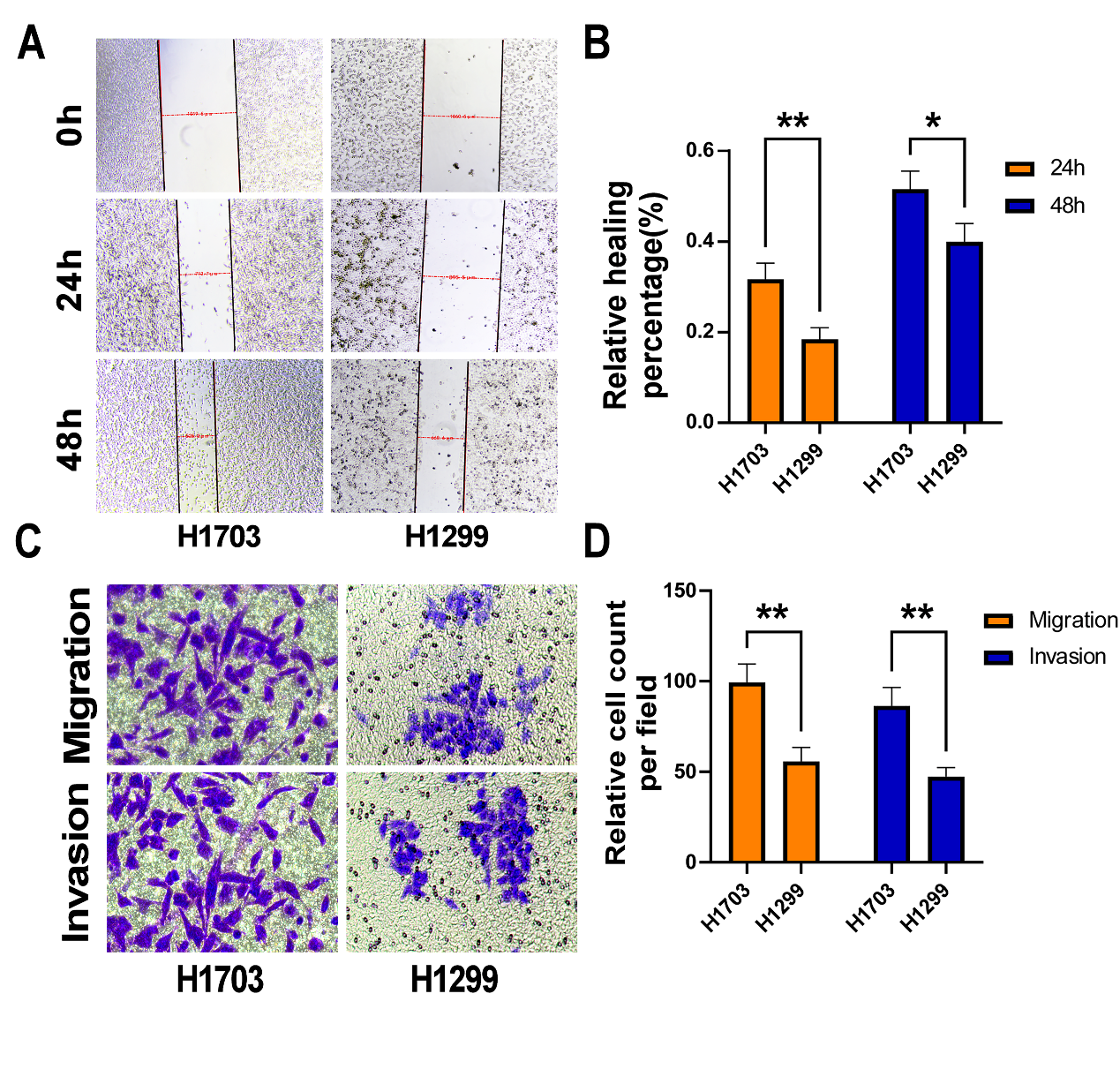


**Fig. S2 H1703 cells had the higher capability in cell migration and invasion than H1299 cells**

(A, B) Wound healing assay was performed to detect migration of H1703 cells and H1299 cells, representative images were taken at 40× magnification at 0, 24 and 48 h. (C, D) Transwell migration and invasion assays were conducted to evaluate the migration and invasion of H1703 cells and H1299 cells. Representative images were taken at 200× magnification. Data are expressed as means ± standard deviation. ^*^*P*<0.05, ^**^*P*<0.01 vs H1703 group.


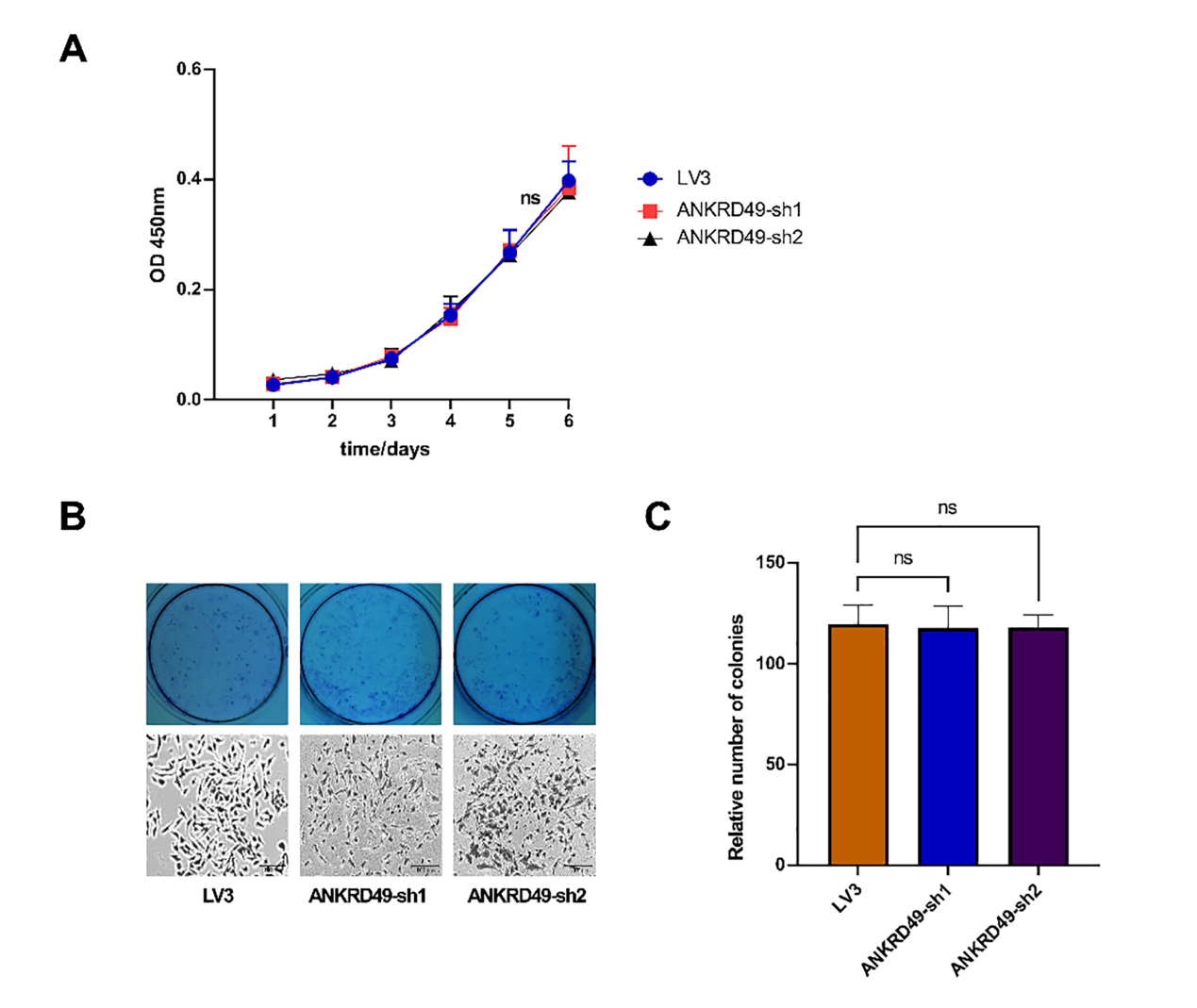


**Fig. S4 ANKRD49 has no effect on cellular proliferation of H1703 cells.**

Proliferation assays were conducted using CCK8 (A) and colony formation (B, C) in ANKRD49-sh H1703 cells. All experiments were repeated independently three times. Data are presented as means ± standard deviation. ns, not significant.


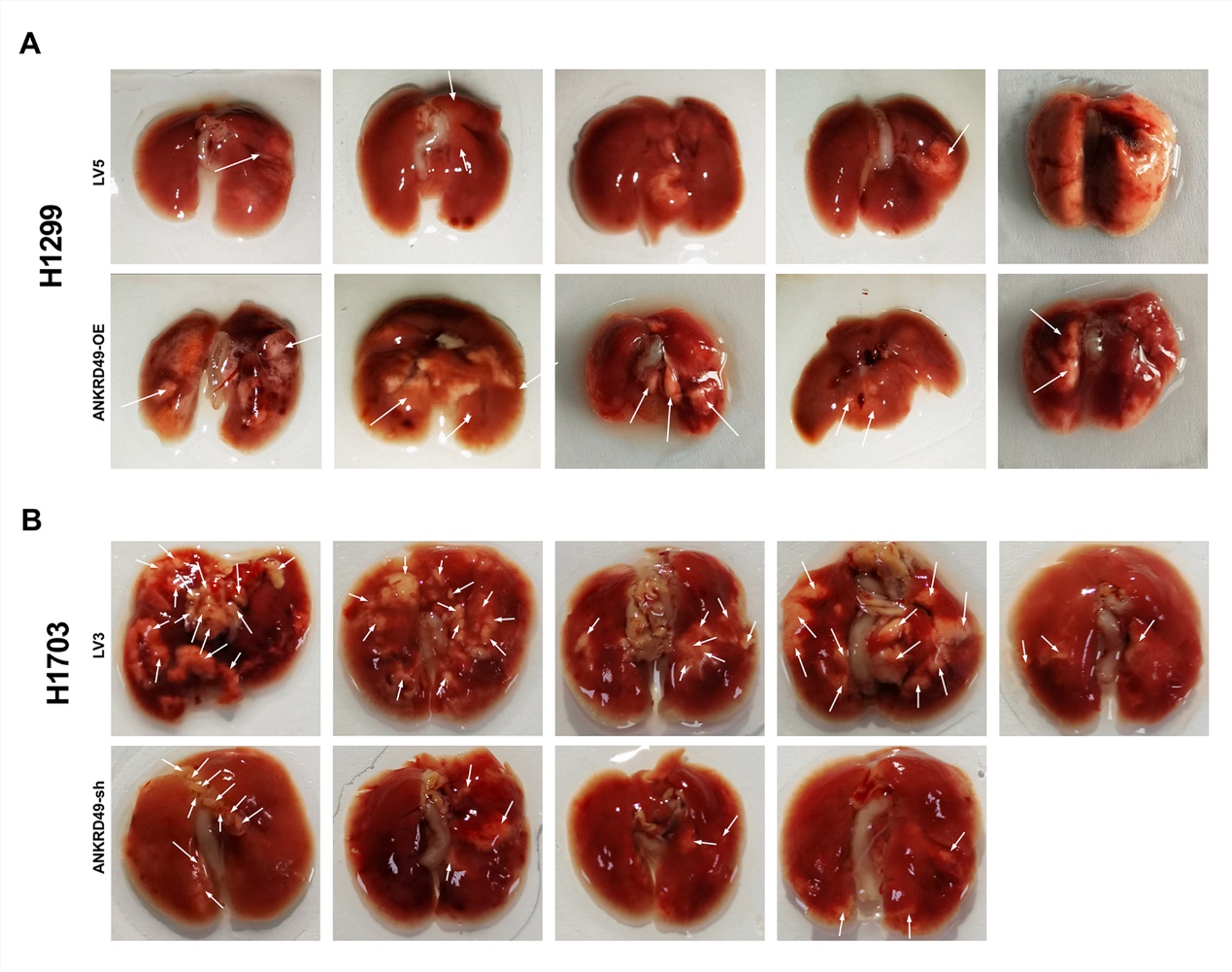


**Fig. S5 ANKRD49 promotes the lung metastasis of NSCLC cells in nude mice.**

(A) Images of the lung metastasis nodules in mice injected with ANKRD49-OE or LV5 H1299 cells. (B) Images of the lung metastasis nodules in mice injected with ANKRD49-sh or LV3 H1703 cells. White arrows indicate the metastasis nodules on the lung.


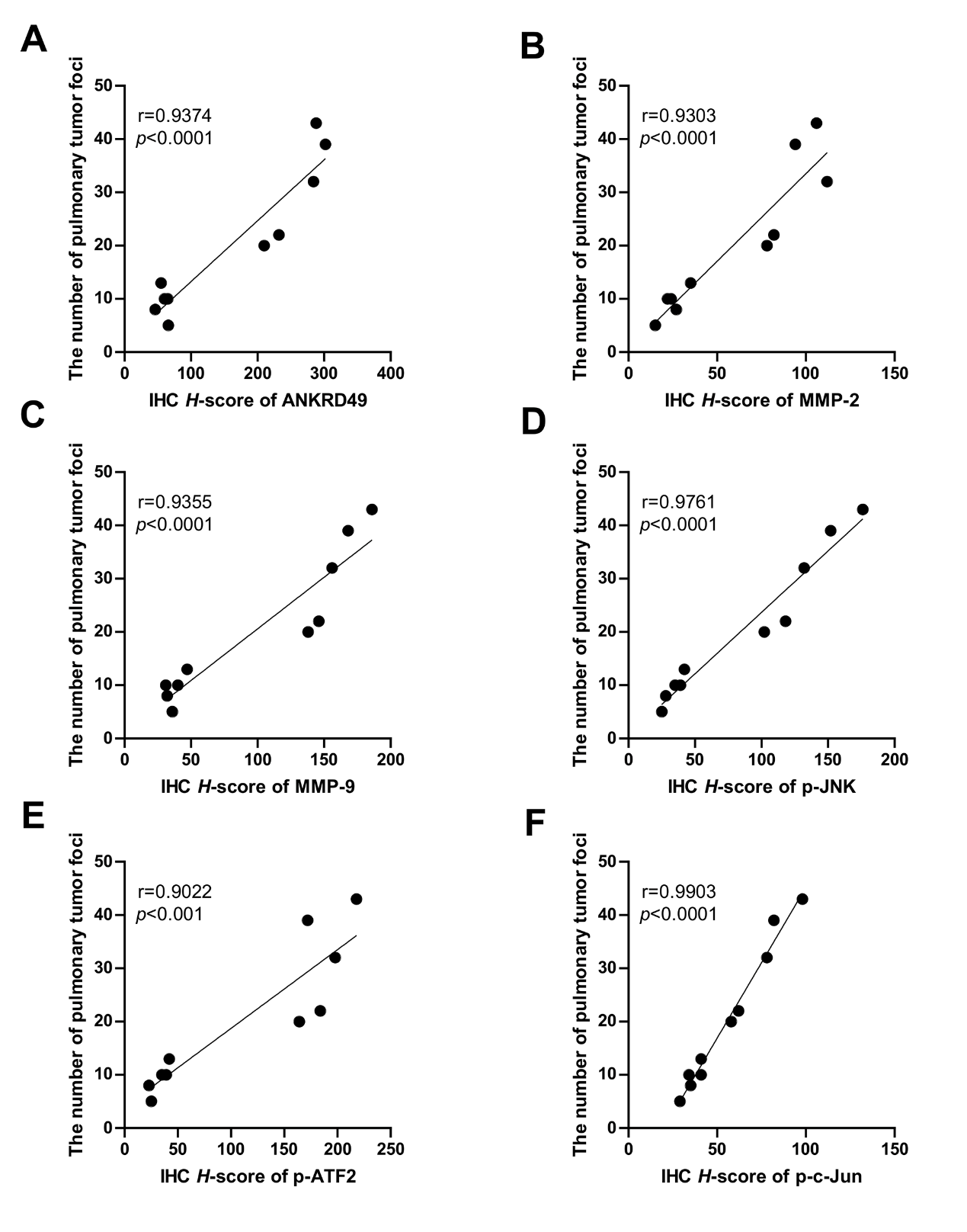


**Fig. S6 Correlation analysis between H-score of ANKRD49, MMP-2, MMP-9, p-JNK, p-ATF2 or p-c-Jun and lung metastasis in mice injected with ANKRD49-OE H1299 cells.**

(A) Correlation analysis between H-score of ANKRD49 and lung metastasis in mice injected with ANKRD49-OE H1299 cells. (B) Correlation analysis between H-score of MMP-2 and lung metastasis in mice injected with ANKRD49-OE H1299 cells. (C) Correlation analysis between H-score of MMP-9 and lung metastasis in mice injected with ANKRD49-OE H1299 cells. (D) Correlation analysis between H-score of p-JNK and lung metastasis in mice injected with ANKRD49-OE H1299 cells. (E) Correlation analysis between H-score of p-ATF2 and lung metastasis in mice injected with ANKRD49-OE H1299 cells. (F) Correlation analysis between H-score of p-c-Jun and lung metastasis in mice injected with ANKRD49-OE H1299 cells.

**
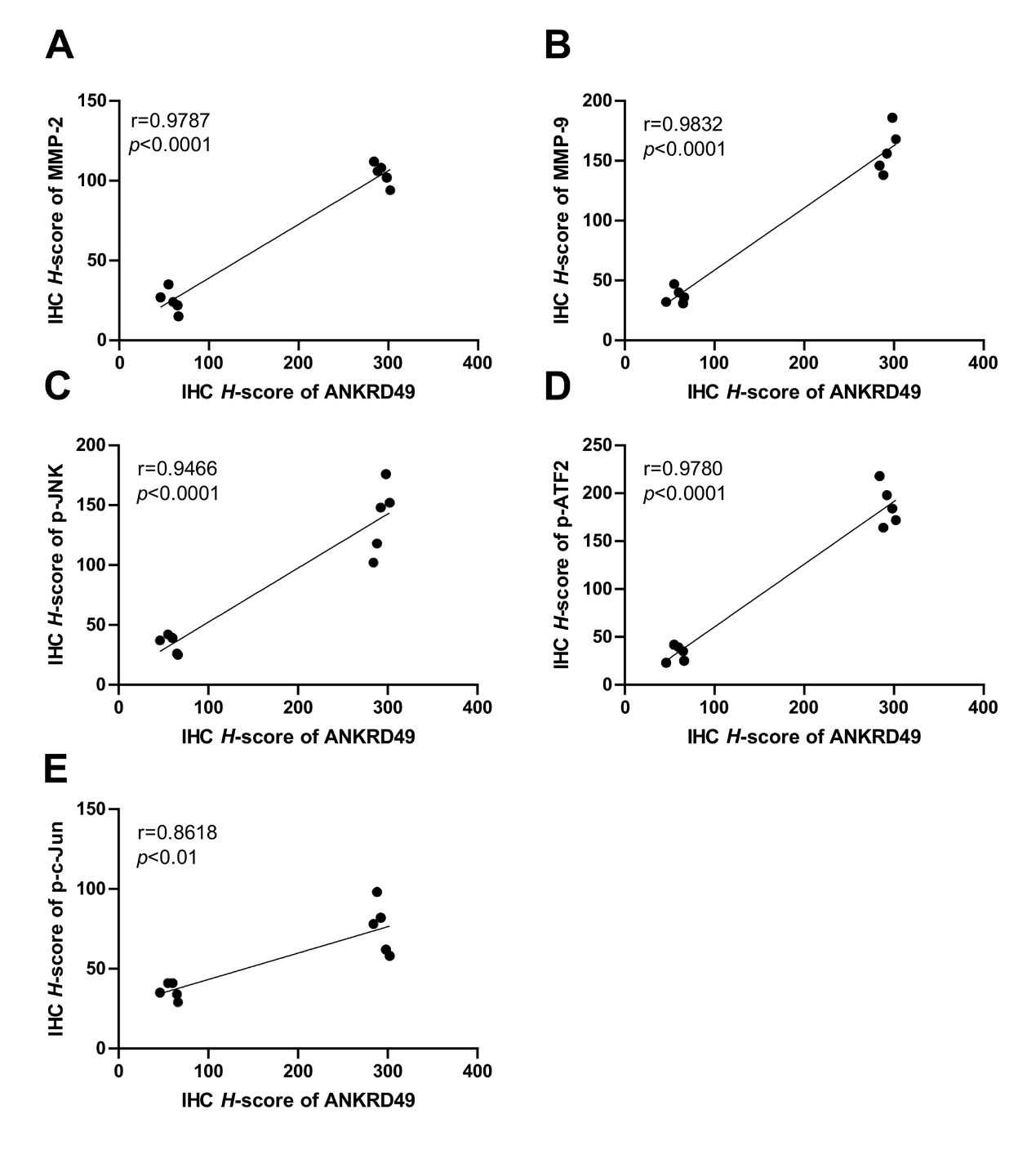
**

**Fig. S7 Correlation analysis between H-score of MMP-2, MMP-9, p-JNK, p-ATF2 or p-c-Jun and ANKRD49 in lung tissues of mice injected with ANKRD49-OE H1299 cells.**

(A) Correlation analysis between H-score of MMP-2 and ANKRD49 in lung tissues of mice injected with ANKRD49-OE H1299 cells. (B) Correlation analysis between H-score of MMP-9 and ANKRD49 in lung tissues of mice injected with ANKRD49-OE H1299 cells. (C) Correlation analysis between H-score of p-JNK and ANKRD49 in lung tissues of mice injected with ANKRD49-OE H1299 cells. (D) Correlation analysis between H-score of p-ATF2 and ANKRD49 in lung tissues of mice injected with ANKRD49-OE H1299 cells. (E) Correlation analysis between H-score of p-c-Jun and ANKRD49 in lung tissues of mice injected with ANKRD49-OE H1299 cells.
