# Supplementary material for: ANKRD49 promotes the metastasis of NSCLC via activating JNK-ATF2/c-Jun-MMP-2/9 axis
Source: BMC Cancer. 2023 Nov 14;23:1108. doi: 10.1186/s12885-023-11612-9 (PMC10644579; doi:10.1186/s12885-023-11612-9)
Supplement: Supplementary file 2 — Additional file 2. Additional Tables [file 12885_2023_11612_MOESM2_ESM.docx]

**Supplementary Tables**

Supplementary Table S1 Target sequences for knockdown of ANKRD49

Supplementary Table S2 Primers used for real time qPCR in gene expression analysis

Supplementary Table 3 Primers used for ChIP assays.

**Supplementary Table S1 Target sequences for knockdown of ANKRD49**

| **Gene** | **sequence** |
| --- | --- |
| LV3-shNC | GTTCTCCGAACGTGTCACGT |
| ANKRD49-sh1 | GCAAGGATACCCTAGAACTCC |
| ANKRD49-sh2 | GGAAGGCTGTACAAATTCTTC |

**Supplementary Table S2 Primers used for real time qPCR in gene expression analysis**

| **Gene** | **Primer sequence** |
| --- | --- |
| ANKRD49 | F: 5′ TGGACACCTTATTCCTACTGG 3′ |
|  | R: 5′ AGTCTCCGCACTGTGGTAA 3′ |
| MMP-2 | F: 5′ TTGACGGTAAGGACGGACTC 3′ |
|  | R: 5′ GGCGTTCCCATACTTCACAC 3′ |
| MMP-9 | F: 5′ GGCGTTCCCATACTTCACAC 3′ |
|  | R: 5′ AGGGACCACAACTCGTCATC 3′ |
| β-actin | F: 5′ CTGGCACCACACCTTCTACA 3′ |
|  | R: 5′ AGCACAGCCTGGATAGCAAC 3′ |

**Supplementary Table S3 Primers used for ChIP assays.**

| **Gene** | **Primer sequence** |
| --- | --- |
| MMP-2 | F: 5′ CAACTTGGCTCTCTGGCTAT 3′ |
|  | R: 5′ AGTGACAAACCTTGGCTGTT 3′ |
| MMP-9 | F:5′ GCCAATAGTGCTAAGTTTGAGC 3′ |
|  | R: 5′ CTGCTACCCACTGAAGGTAAG 3′ |
