# Supplementary material for: ANKRD49 promotes the metastasis of NSCLC via activating JNK-ATF2/c-Jun-MMP-2/9 axis
Source: BMC Cancer. 2023 Nov 14;23:1108. doi: 10.1186/s12885-023-11612-9 (PMC10644579; doi:10.1186/s12885-023-11612-9)
Supplement: Supplementary file 3 — Additional file 3. Original Data [file 12885_2023_11612_MOESM3_ESM.pdf]

Figure 2B

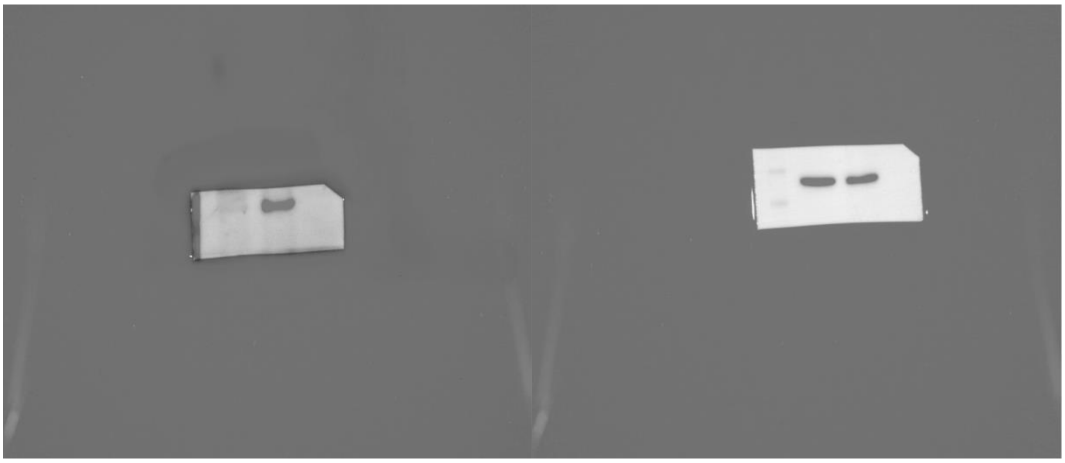

ANKRD49 β-actin

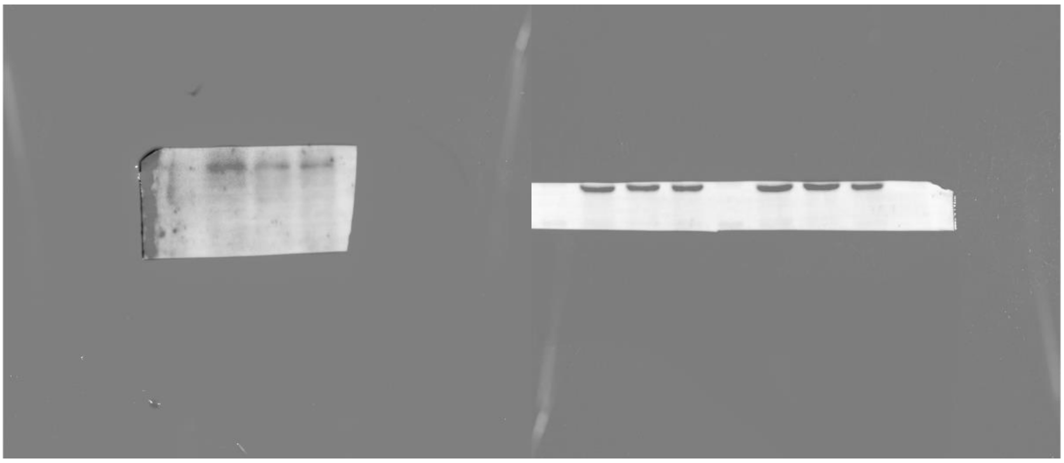

ANKRD49 β-actin

Figure 3B

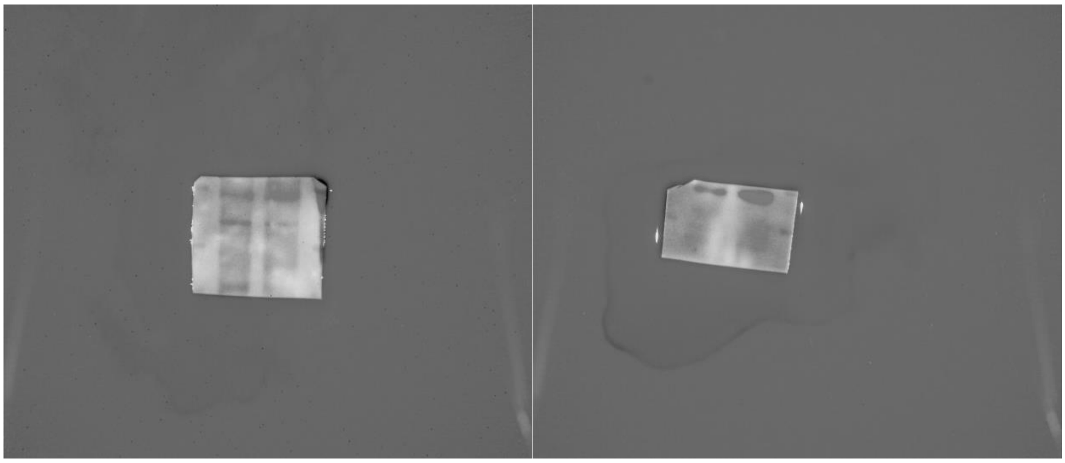

MMP-9 MMP-2

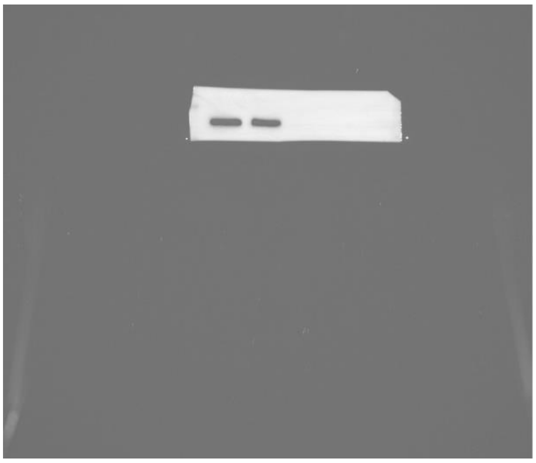

β-actin

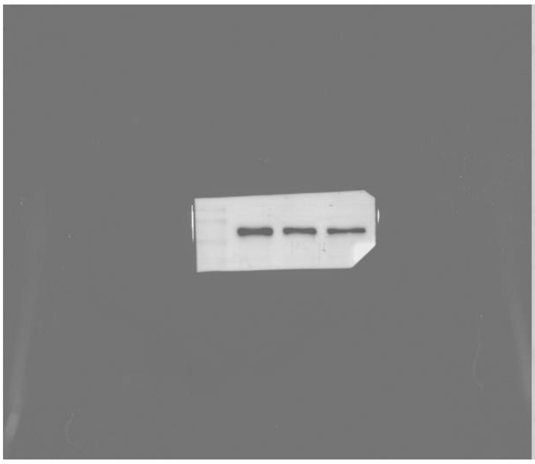

MMP-9

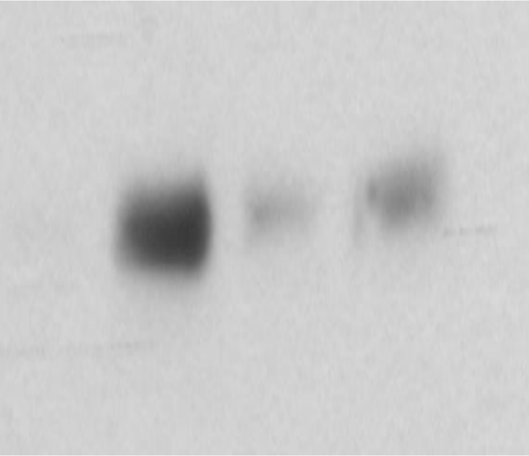

MMP-2

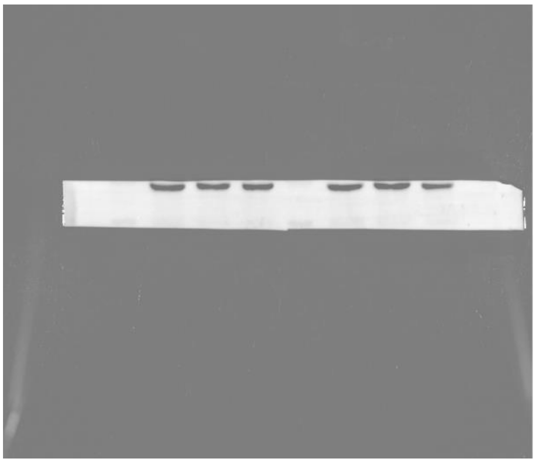

β-actin

Figure 3C

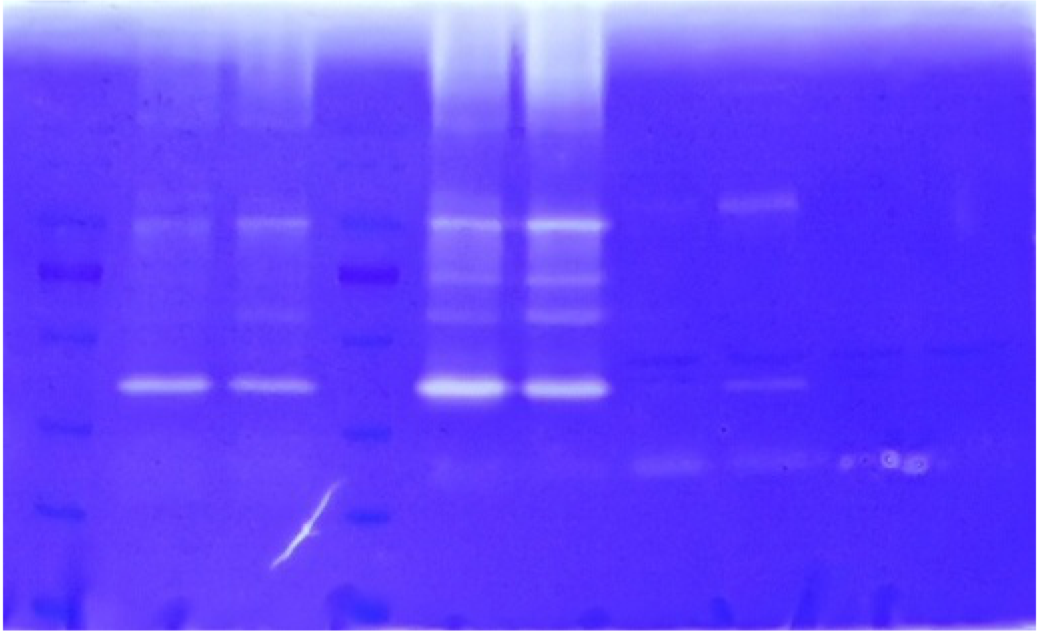

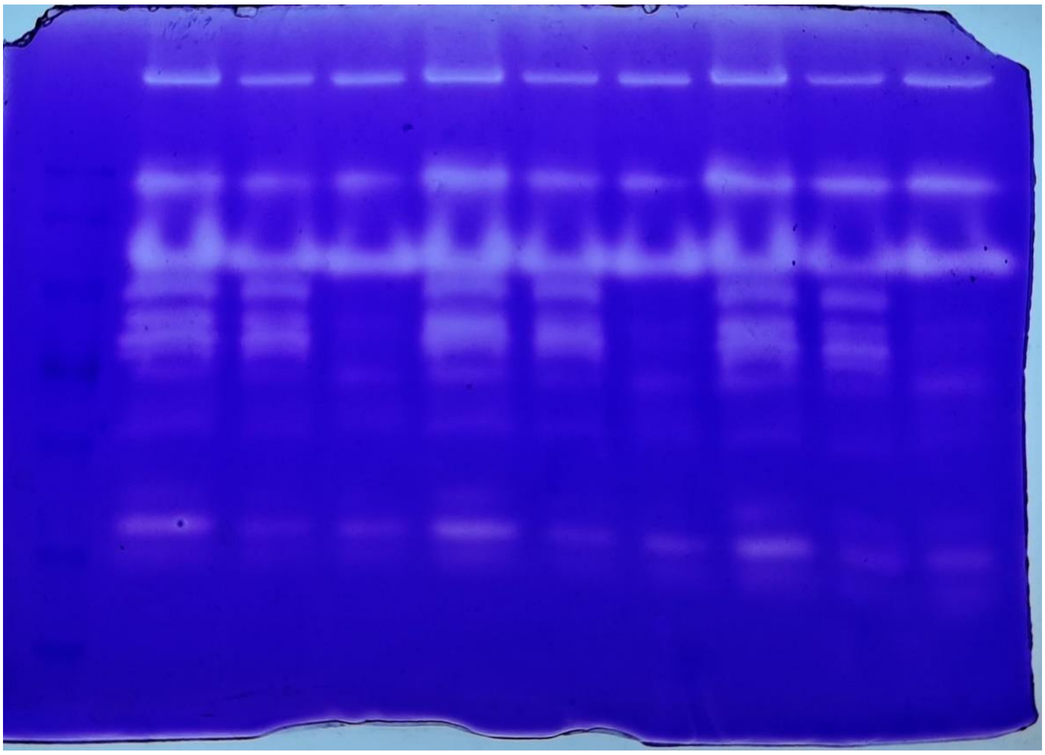

Figure 4A

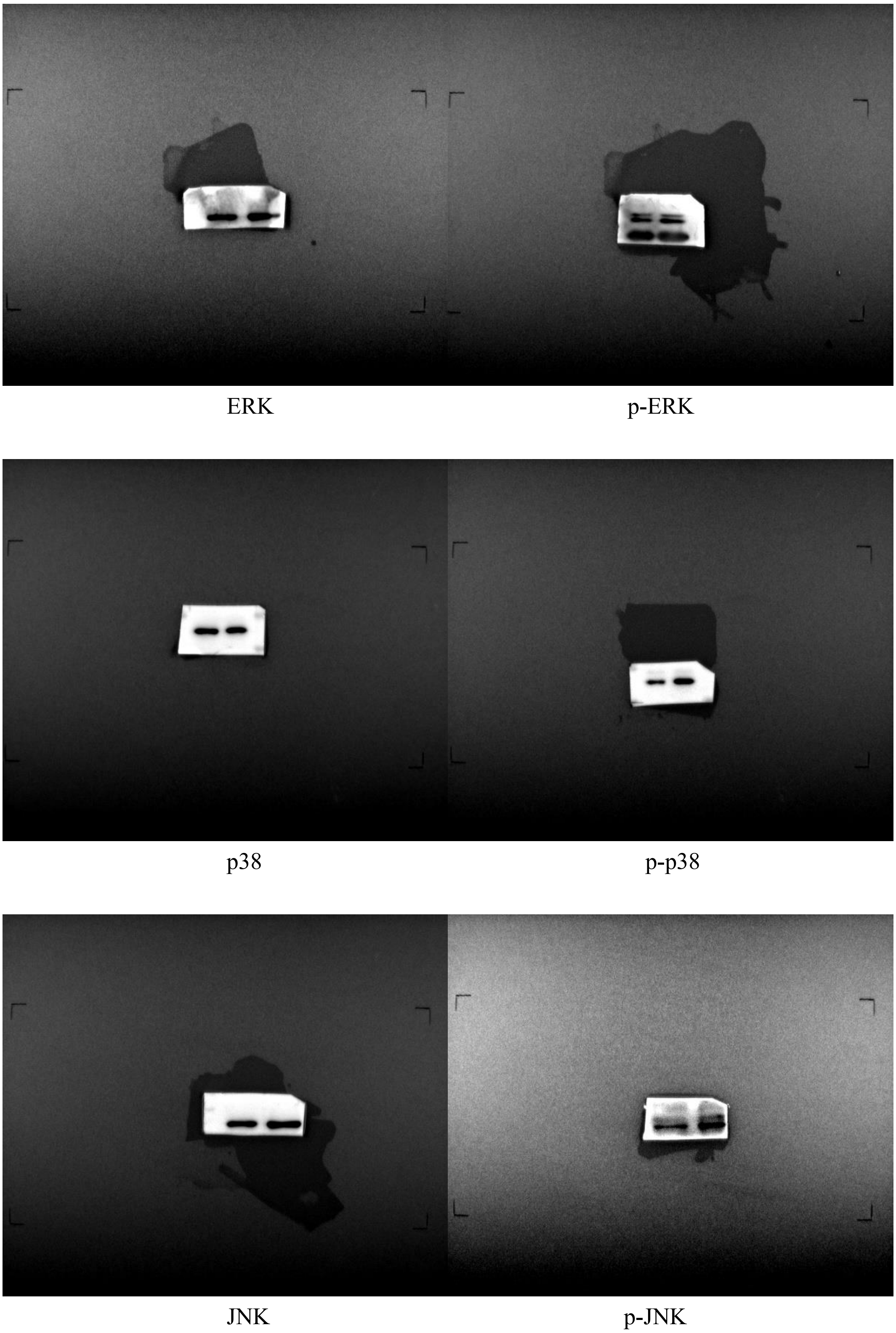

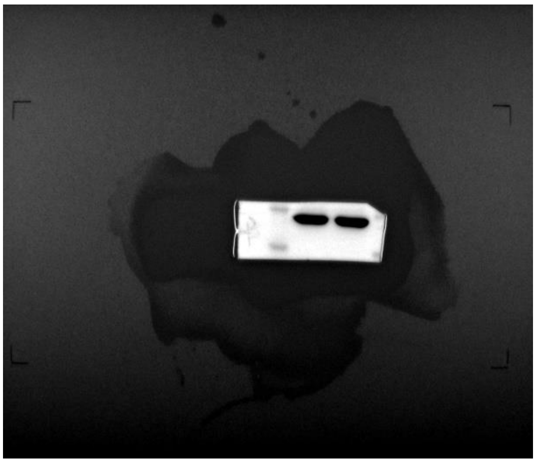

β-Tubulin

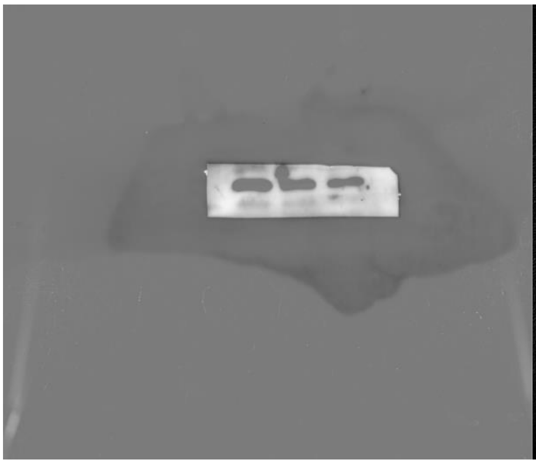

p38

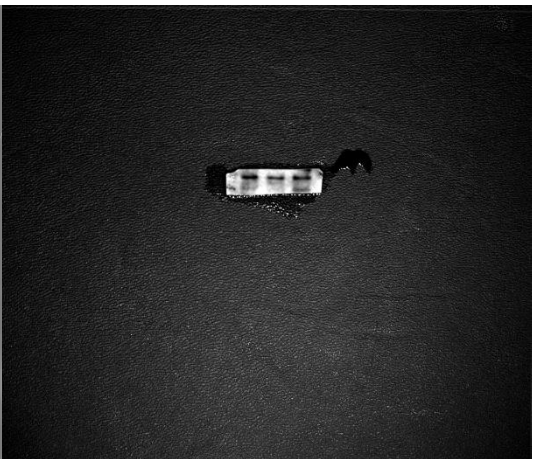

p-p38

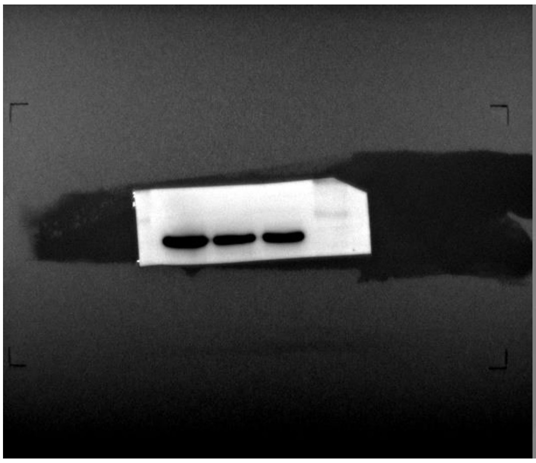

JNK

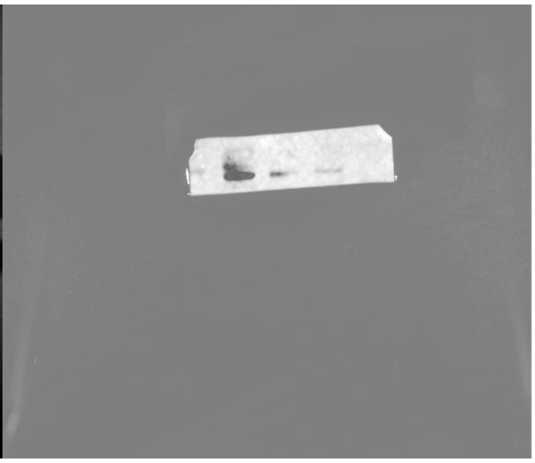

p-JNK

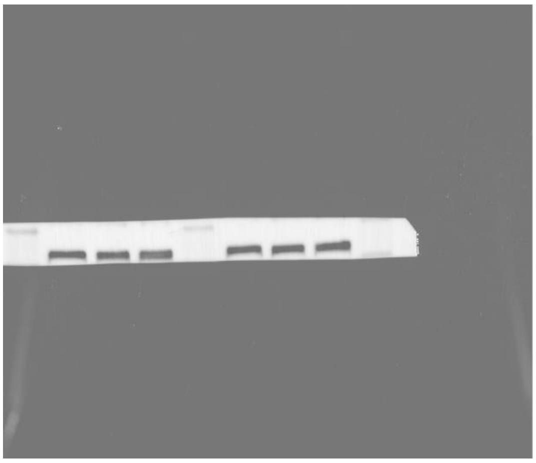

β-Tubulin

Figure 4B

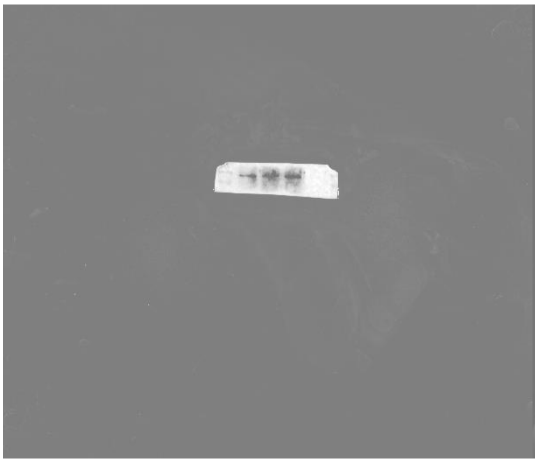

MMP-9

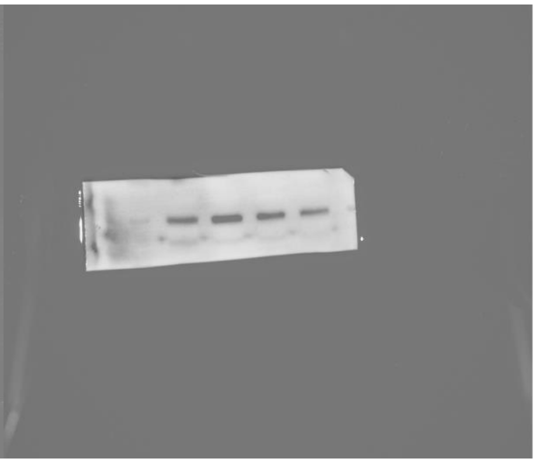

MMP-2

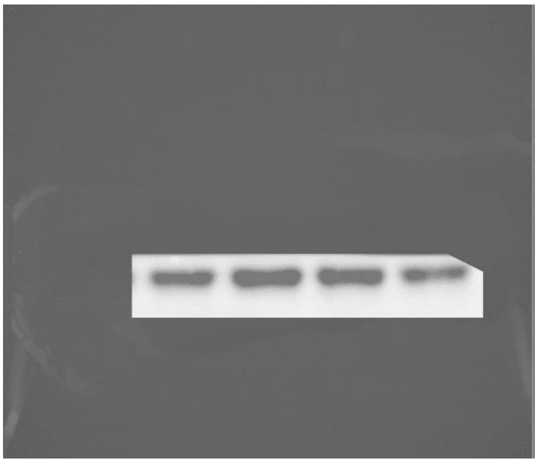

JNK

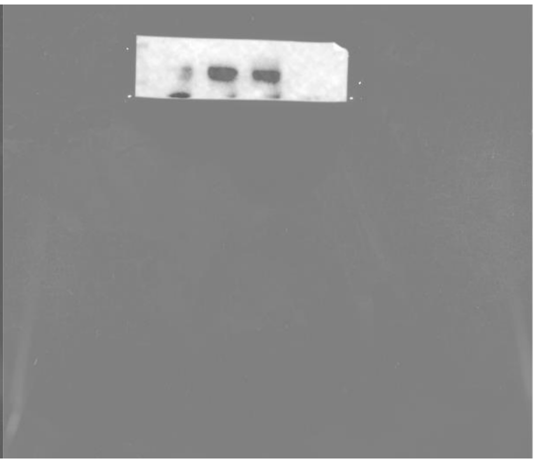

p-JNK

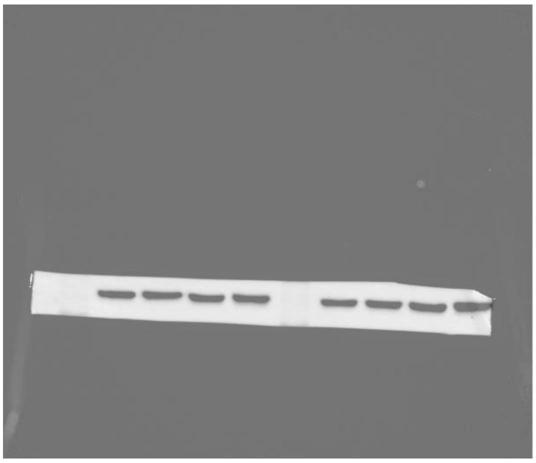

β-Tubulin

Figure 4C

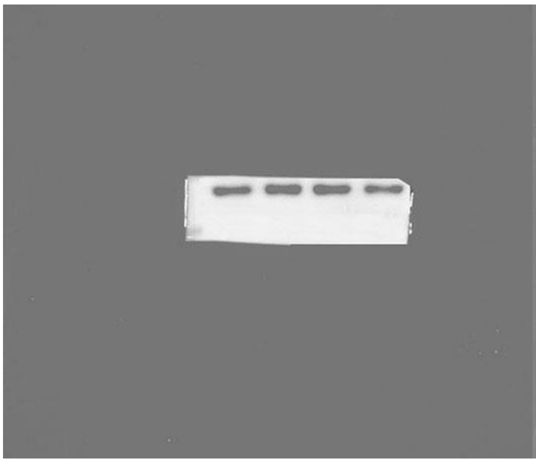

p38

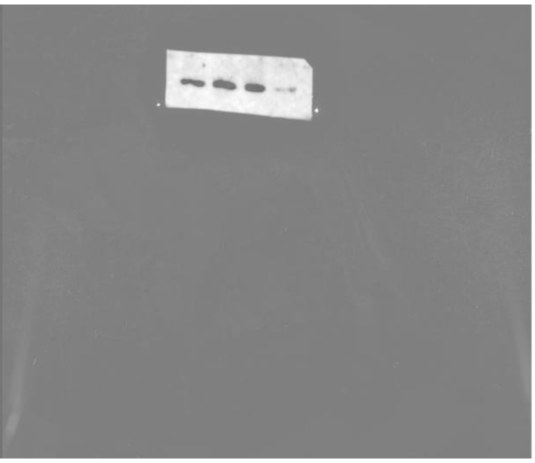

p-p38

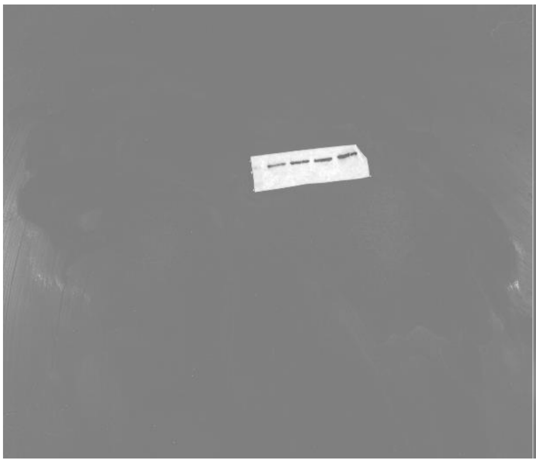

MMP-9

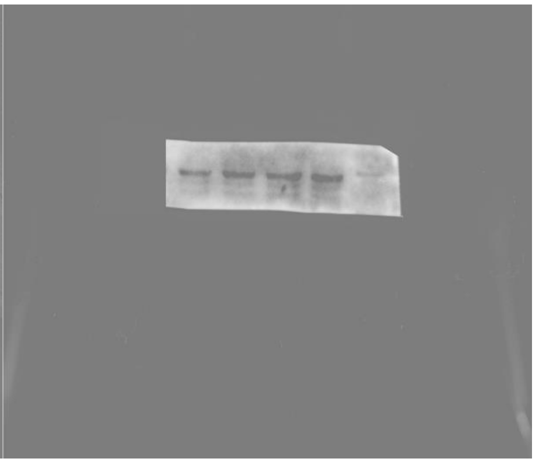

MMP-2

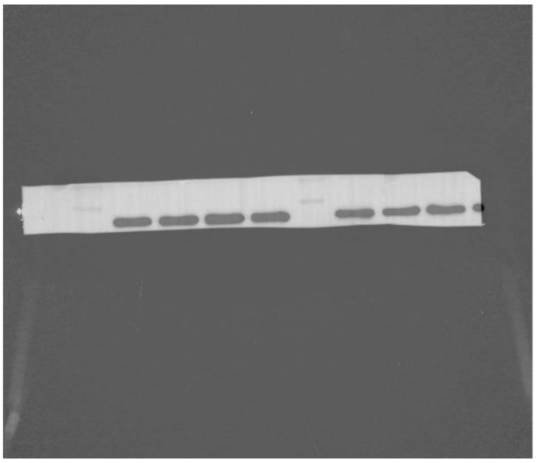

β-Tubulin

Figure 4F

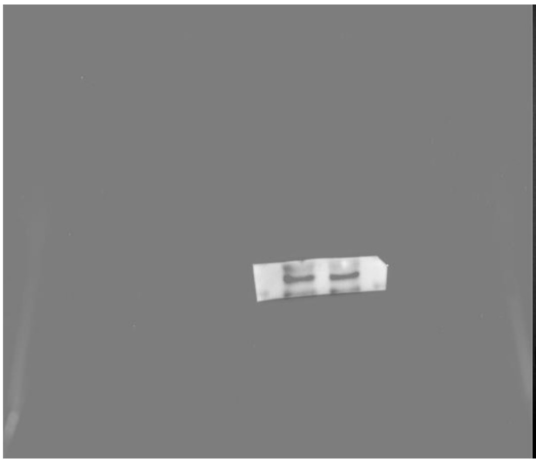

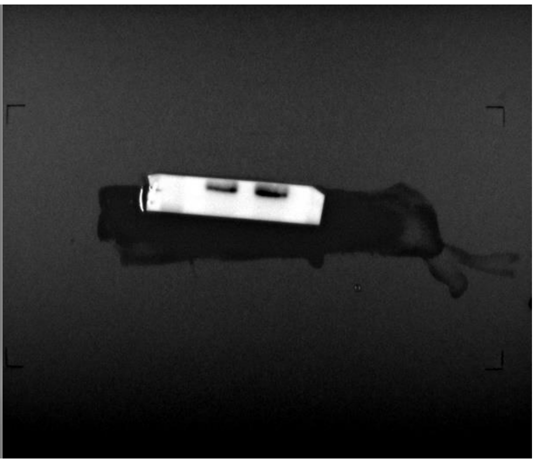

c-Jun p-c-Jun

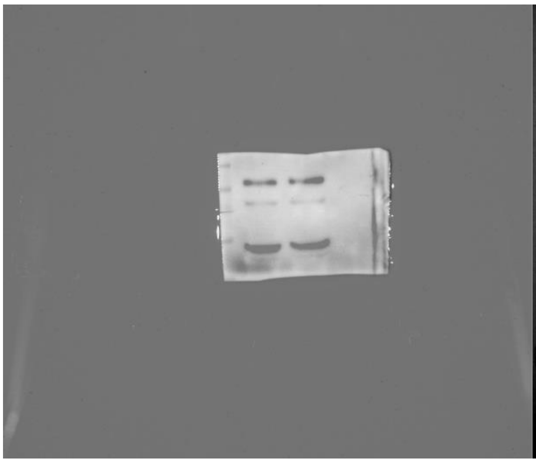

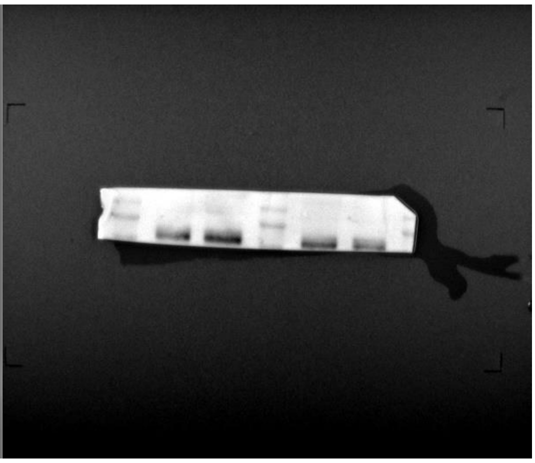

ATF2 p-ATF2

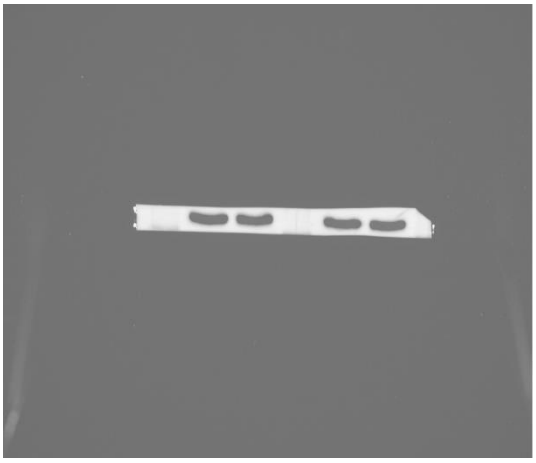

β-Tubulin

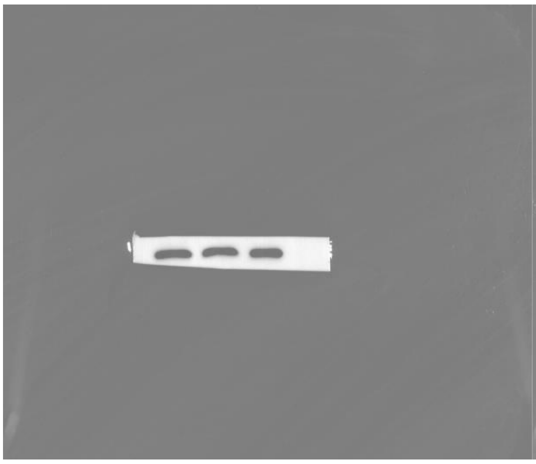

c-Jun

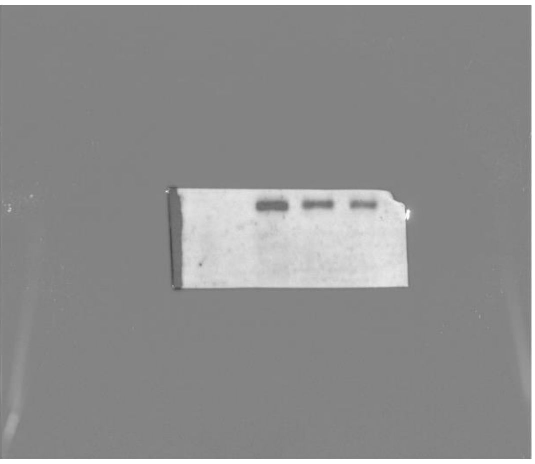

p-c-Jun

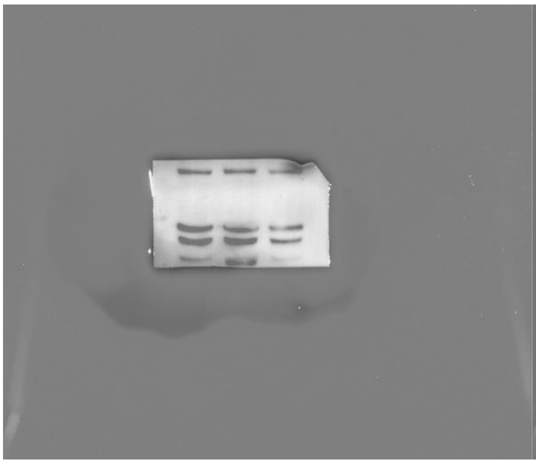

ATF2

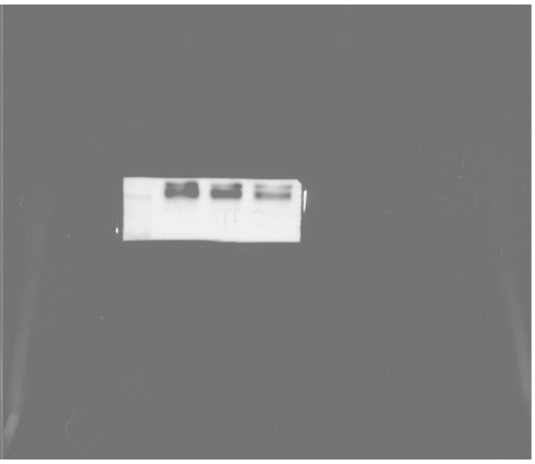

p-ATF2

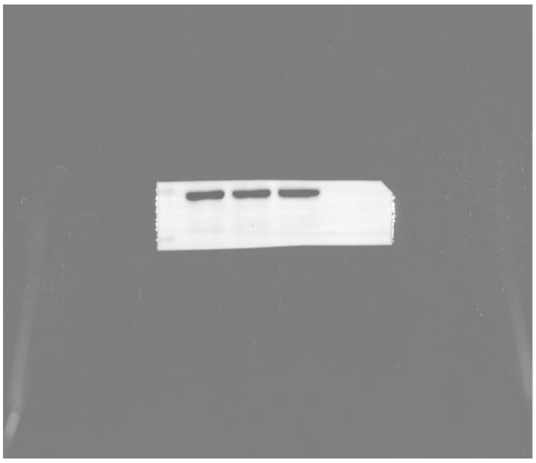

β-Tubulin

Figure 4G

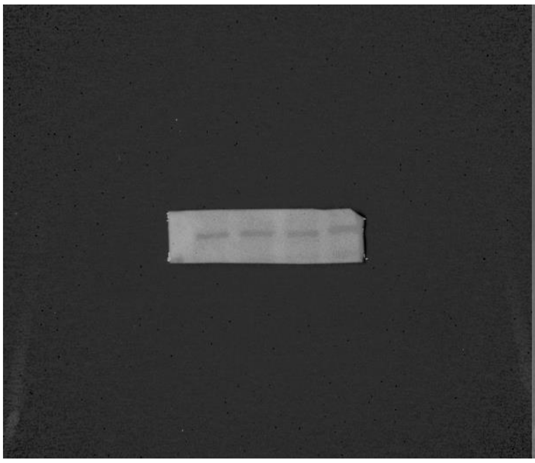

c-Jun

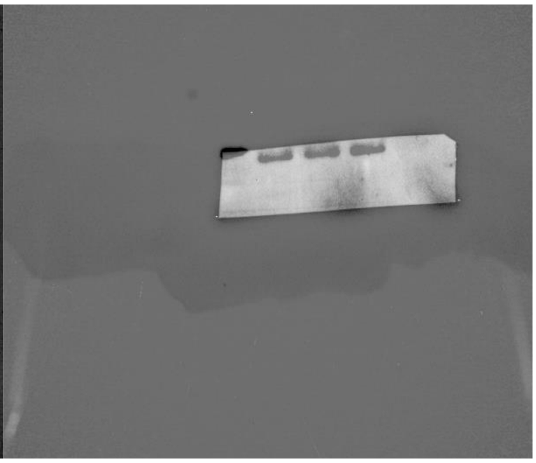

p-c-Jun

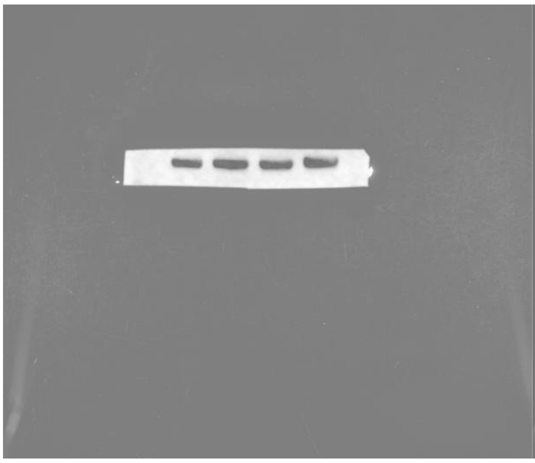

ATF2

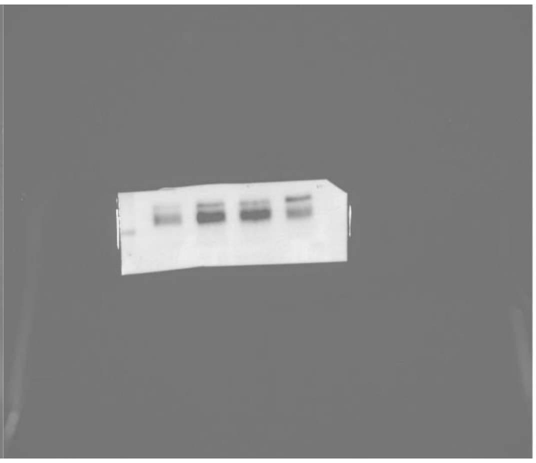

p-ATF2

Figure 4G

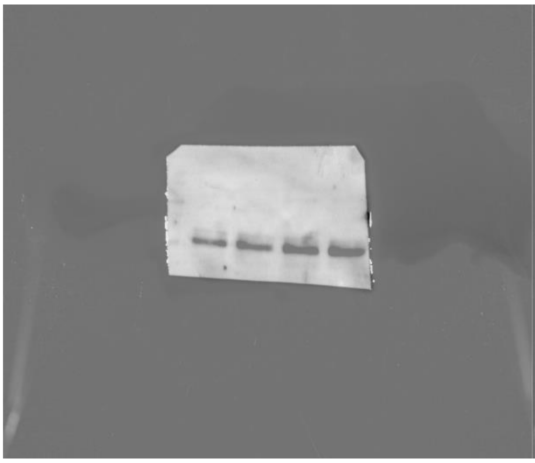

ATF2

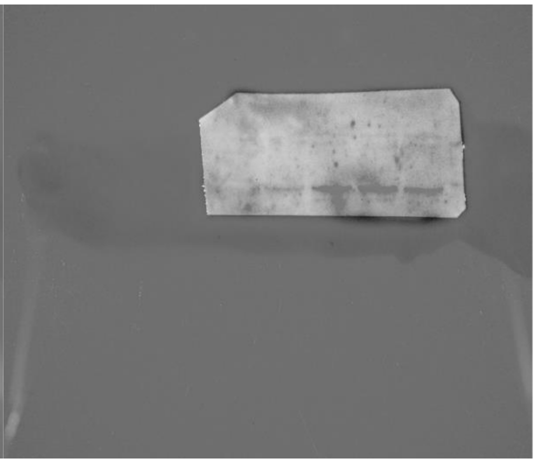

p-ATF2

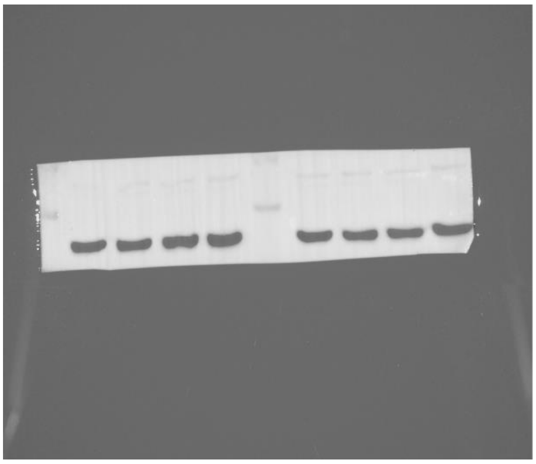

β-Tubulin

Figure 5A

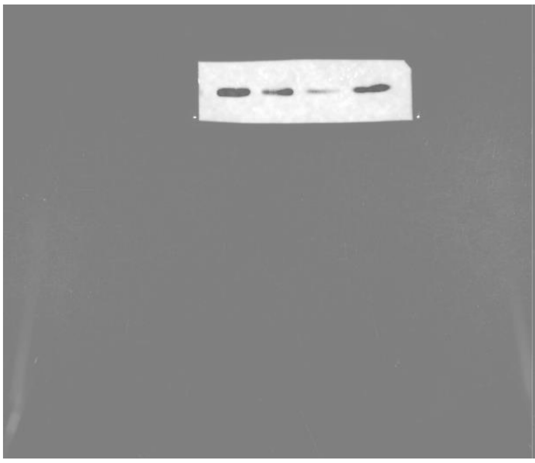

c-Jun

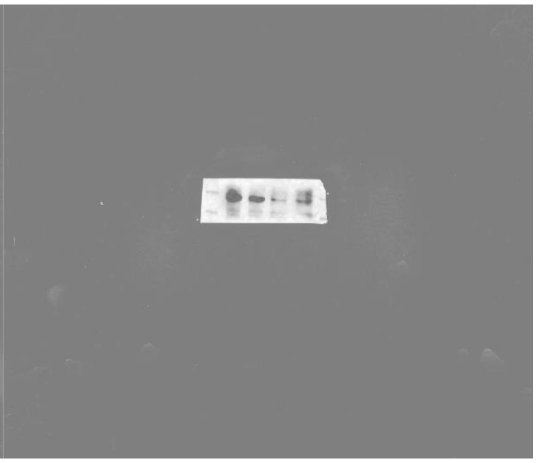

ATF2

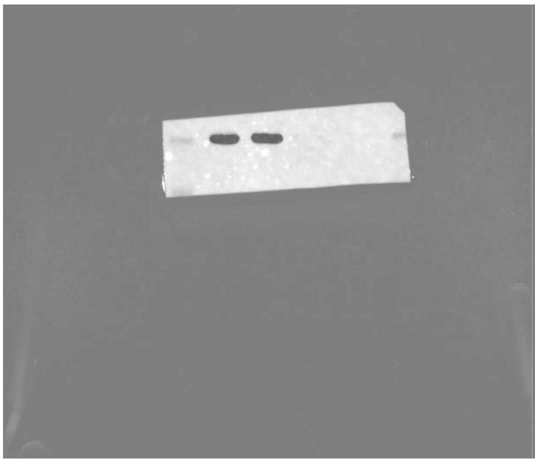

GAPDH

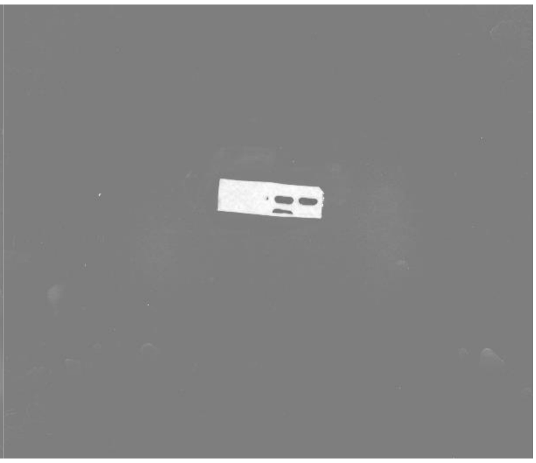

Histone-H3

Figure 5B

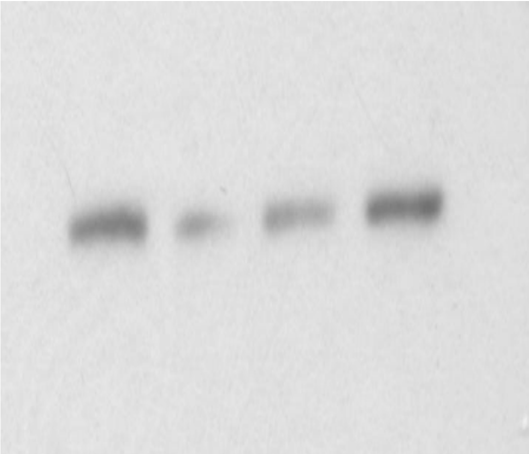

p-c-Jun

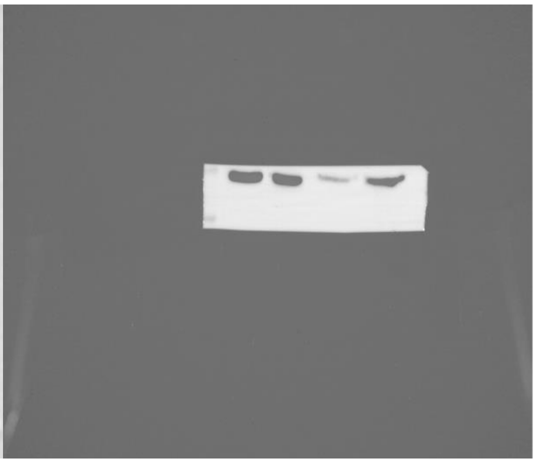

p-ATF2

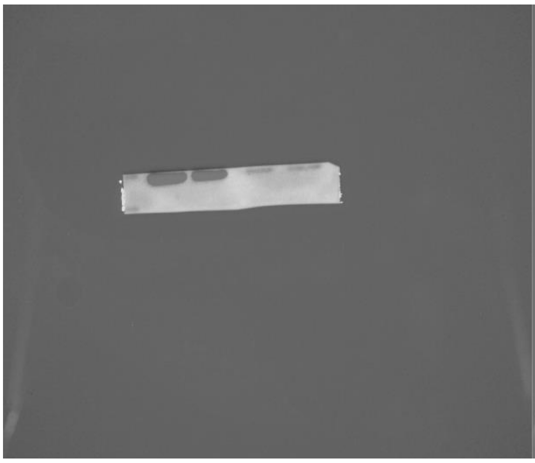

β-actin

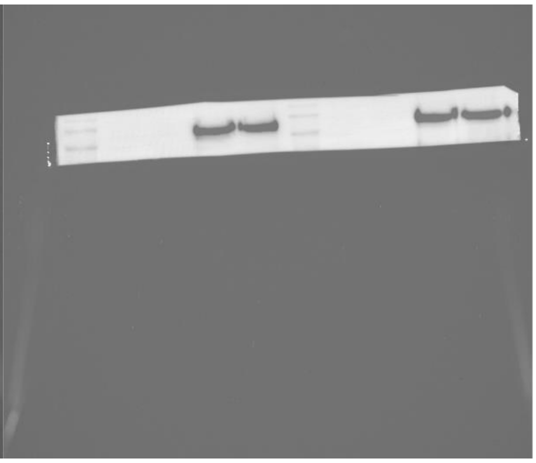

PARP

Figure 5C

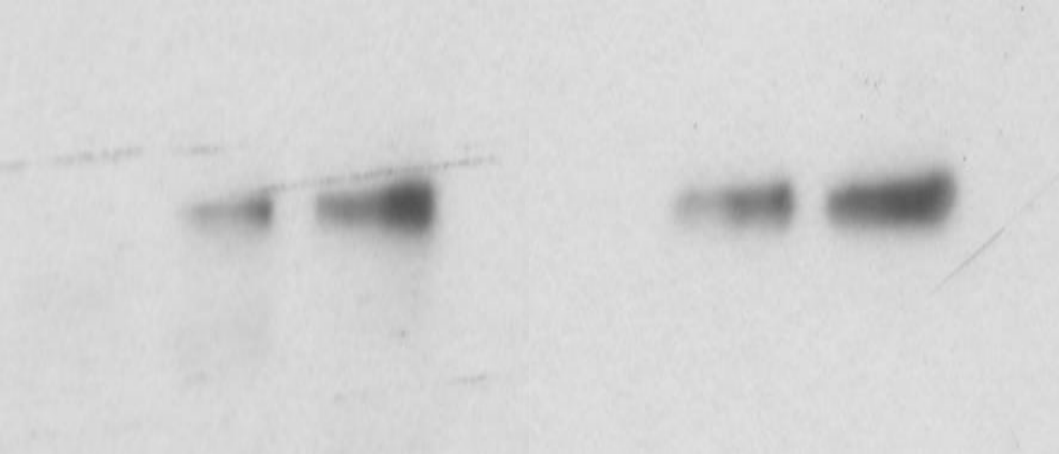

Input p-ATF2 Input p-c-Jun

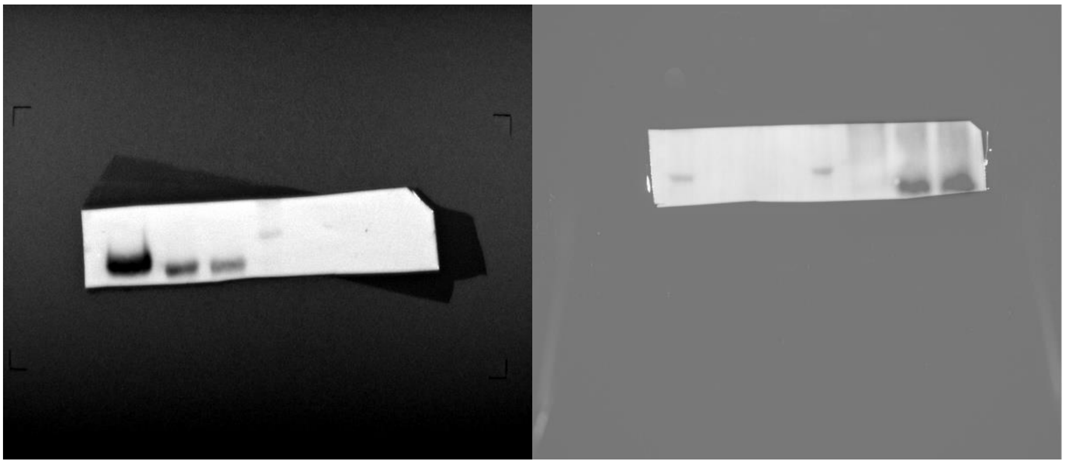

Ip p-ATF2 Ip p-c-Jun

Figure 5E

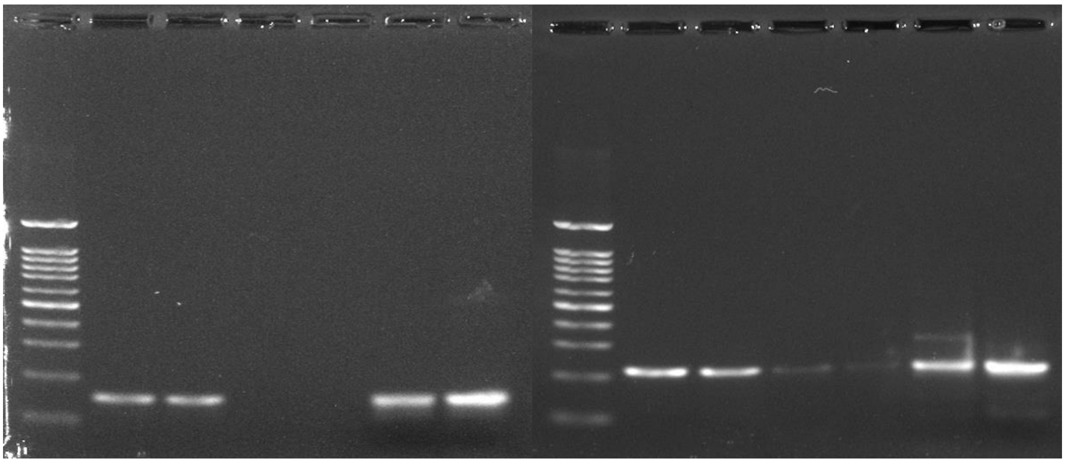

p-ATF2-MMP2 p-ATF2-MMP9

Figure 5G

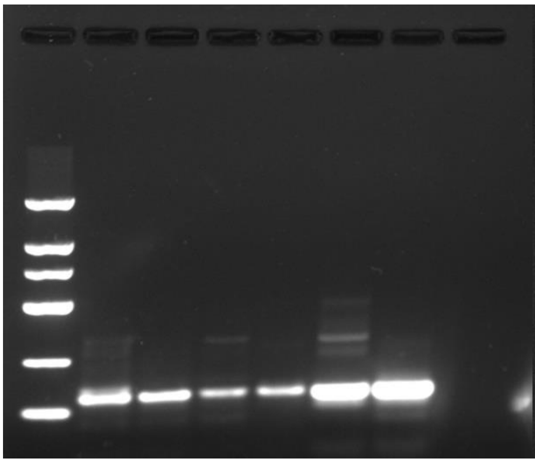

p-c-Jun-MMP2

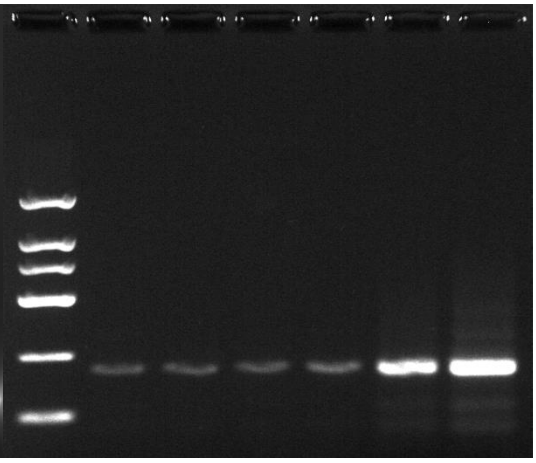

p-c-Jun -MMP9

Figure 6B

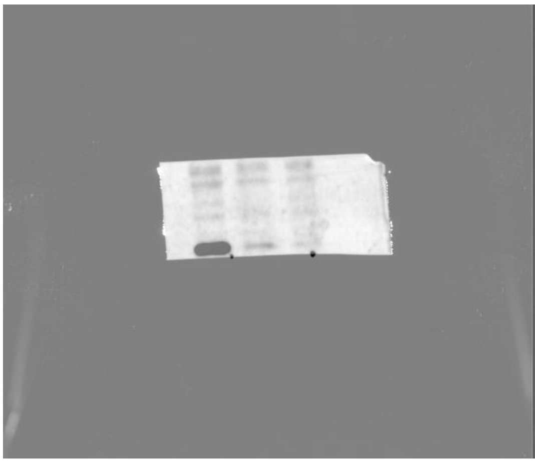

ANKRD49

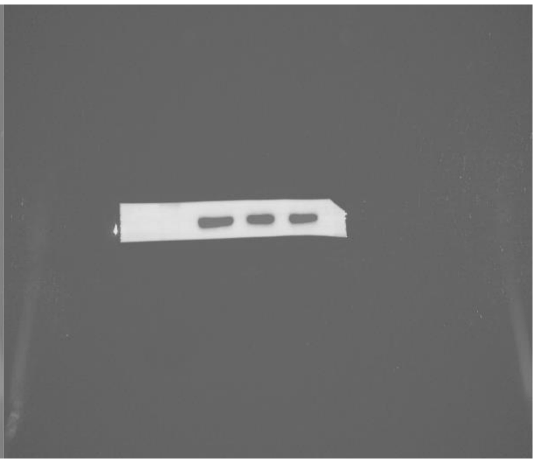

β-actin

Figure 7B

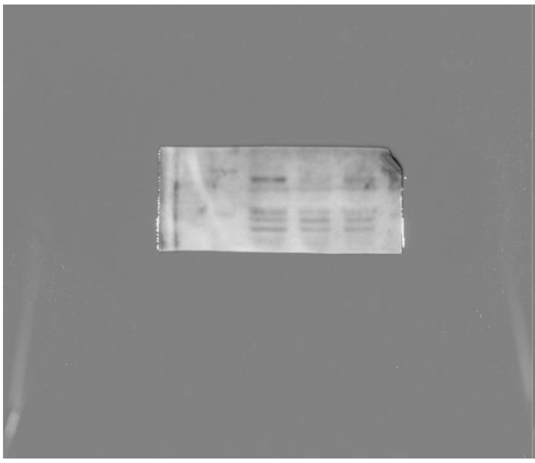

MMP-9

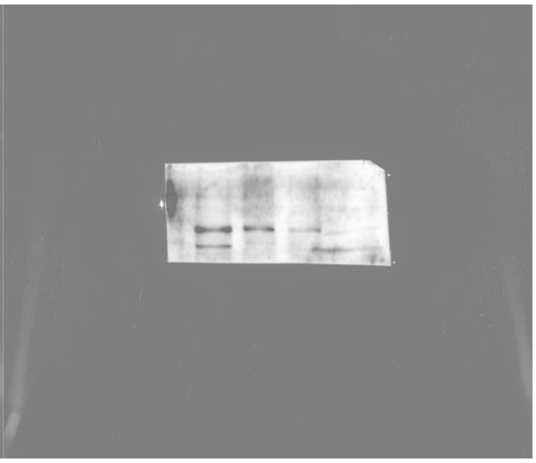

MMP-2

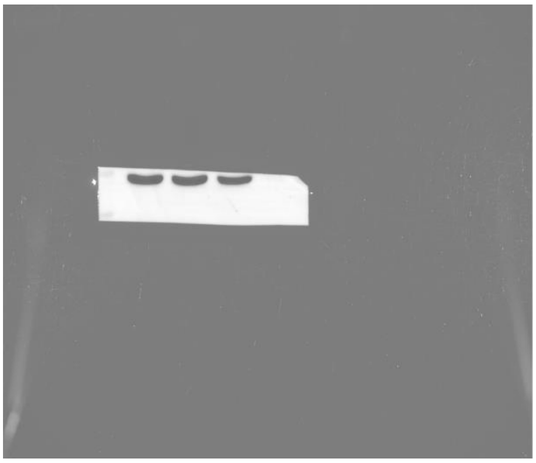

β-actin

Figure 7C

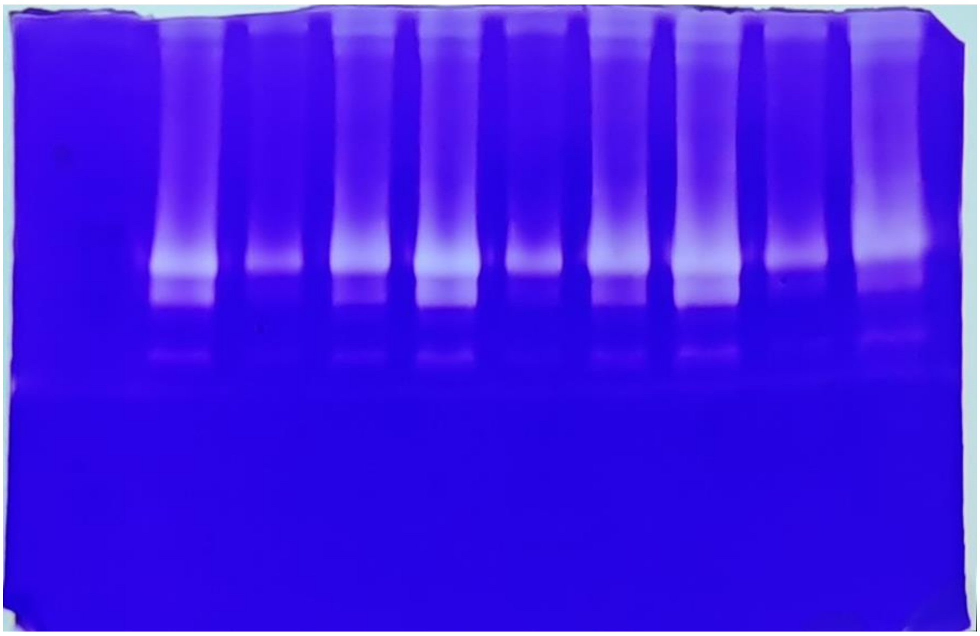

Figure 7E

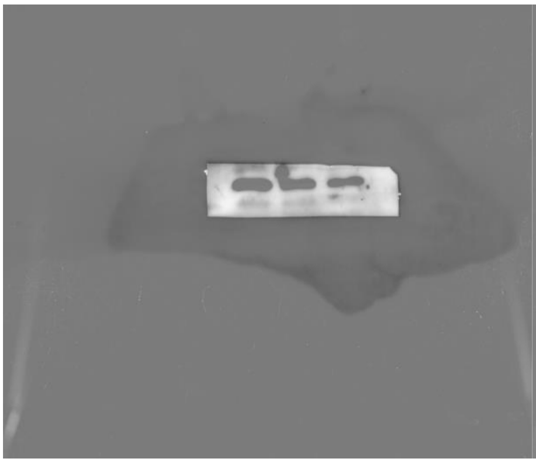

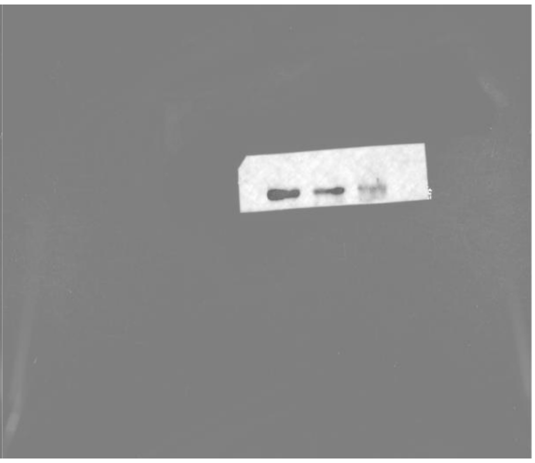

JNK p-JNK

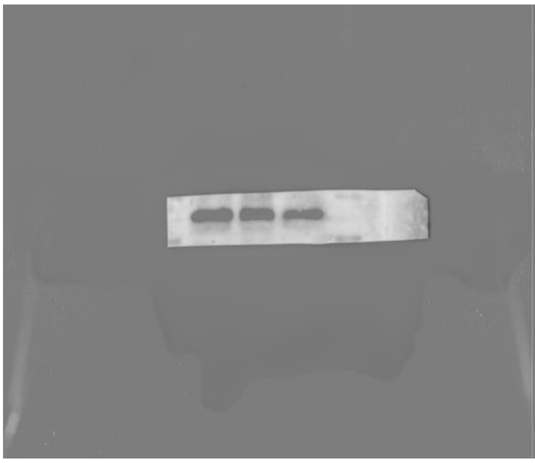

p38

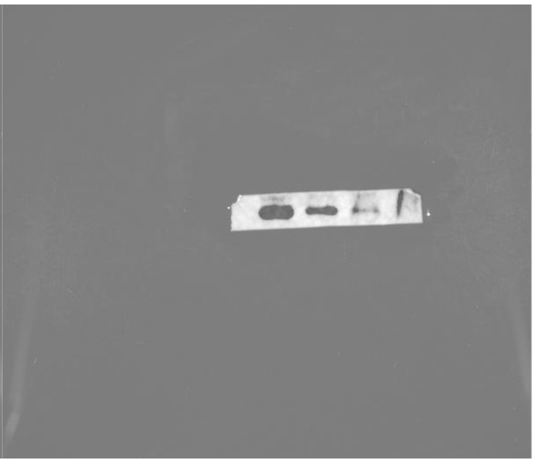

p-p38

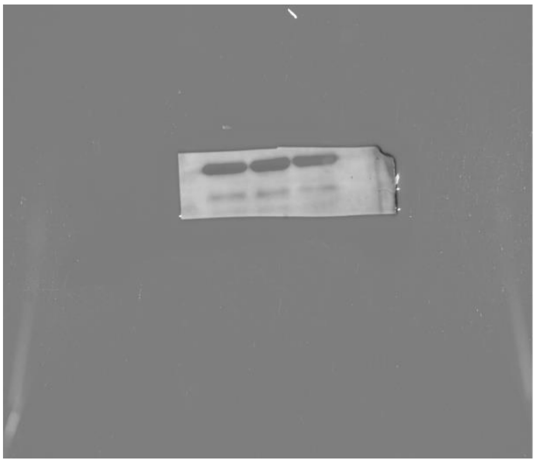

GAPDH

Figure 7F

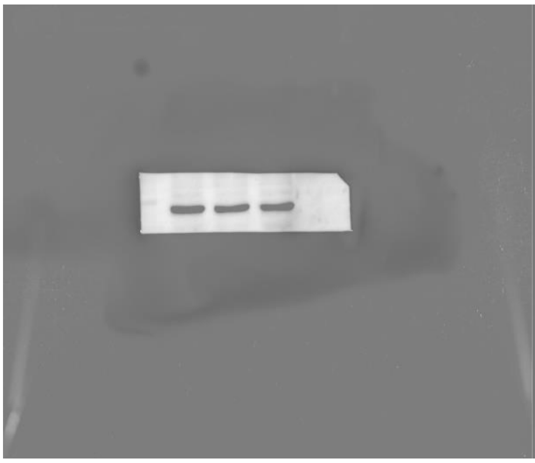

ATF2

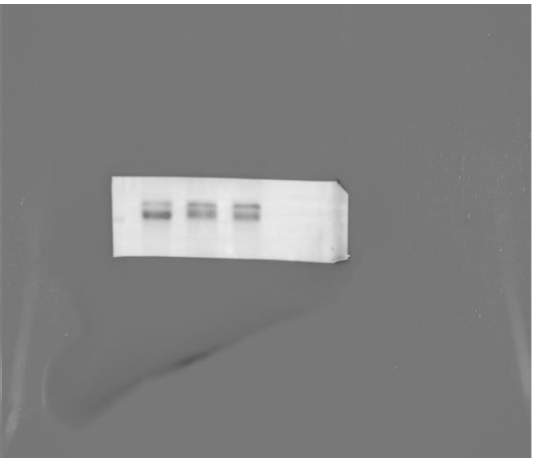

p-ATF2

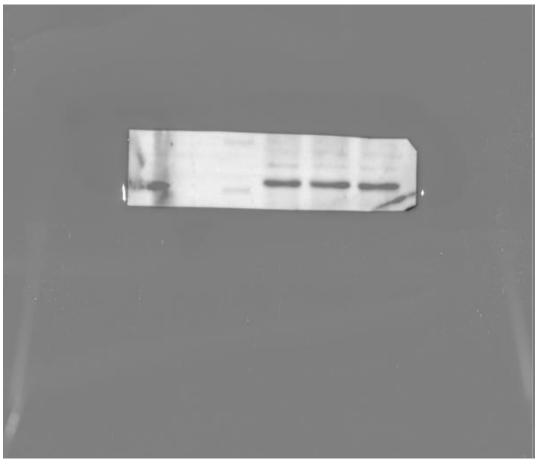

c-Jun

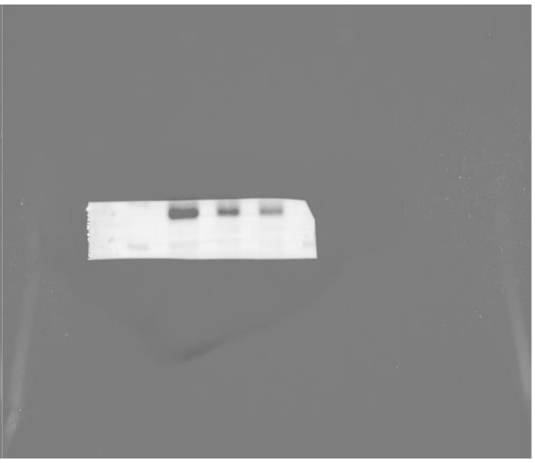

p-c-Jun

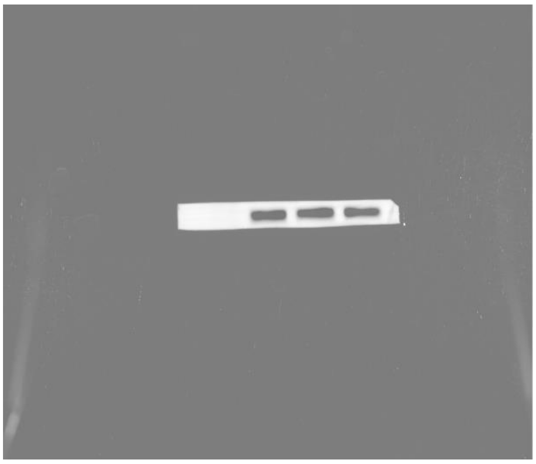

β-Tubulin

Figure 7G

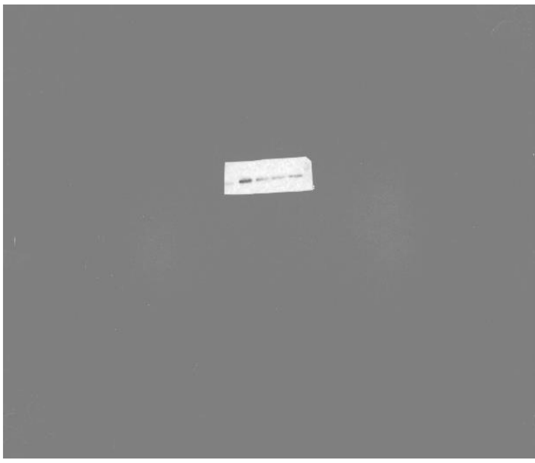

p-p38

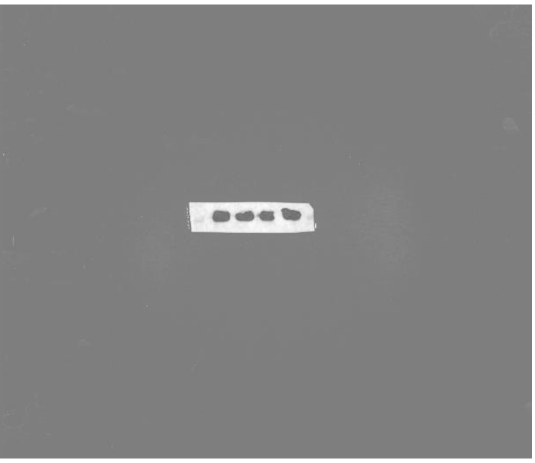

p38

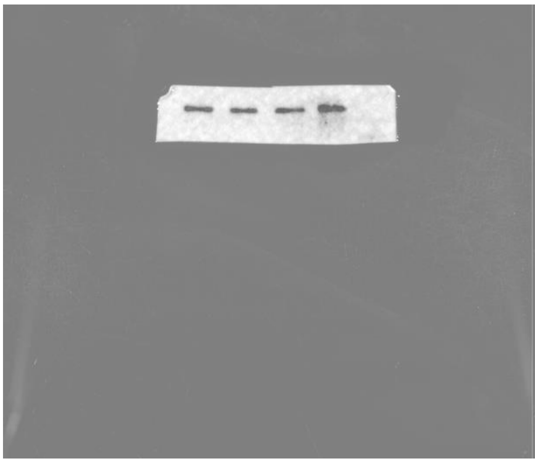

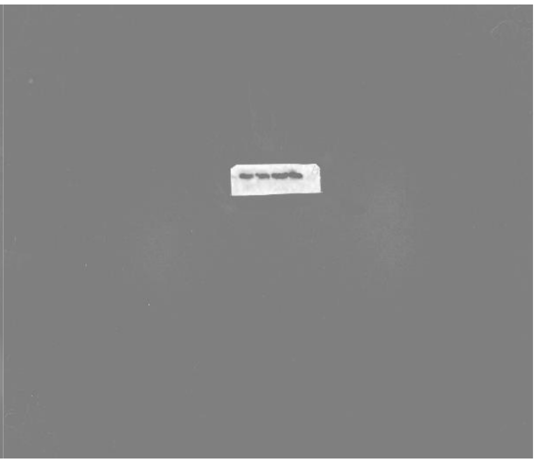

p-JNK JNK

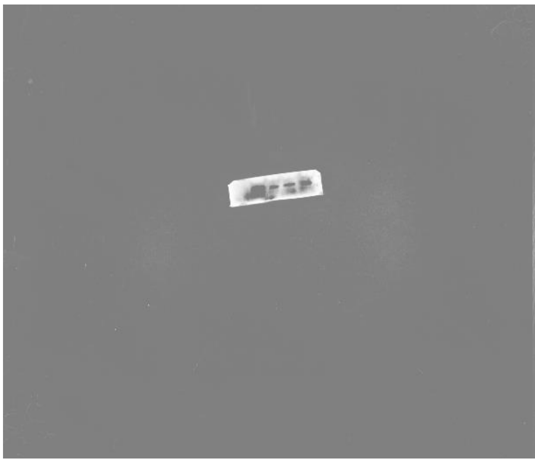

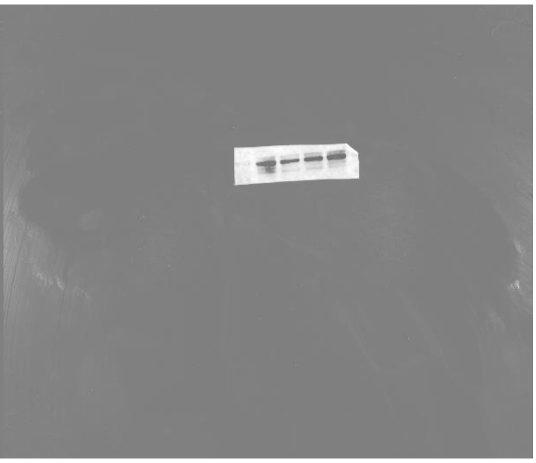

MMP-2 MMP-9

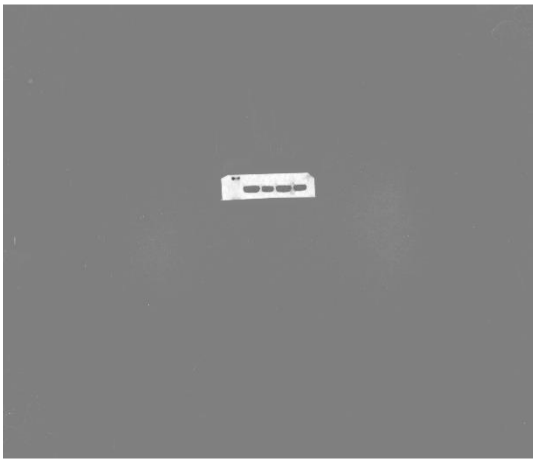

β-Tubulin

Figure 7H

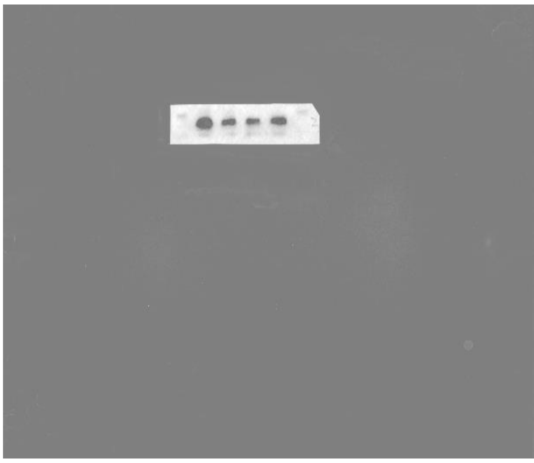

p-p38

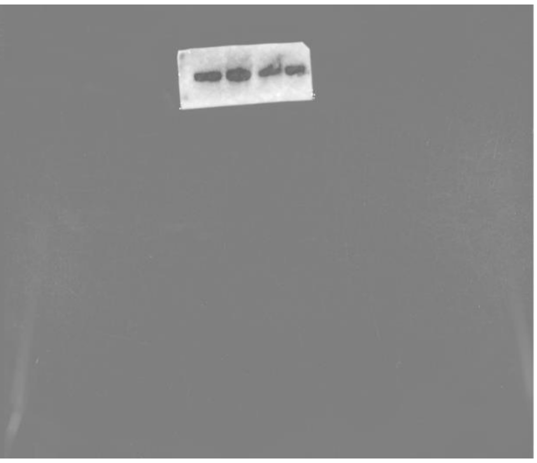

p38

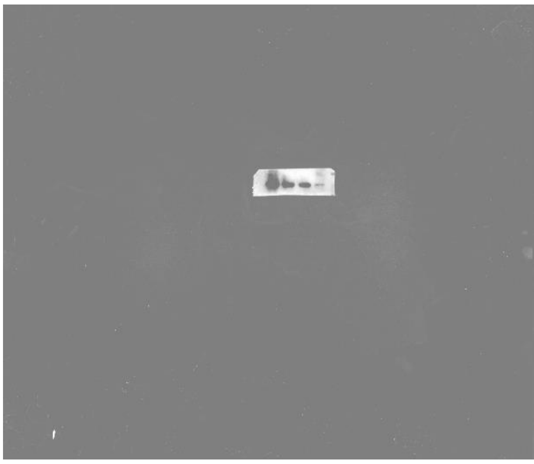

p-JNK

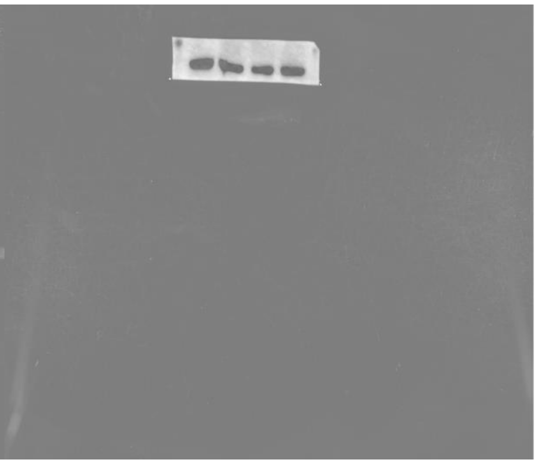

JNK

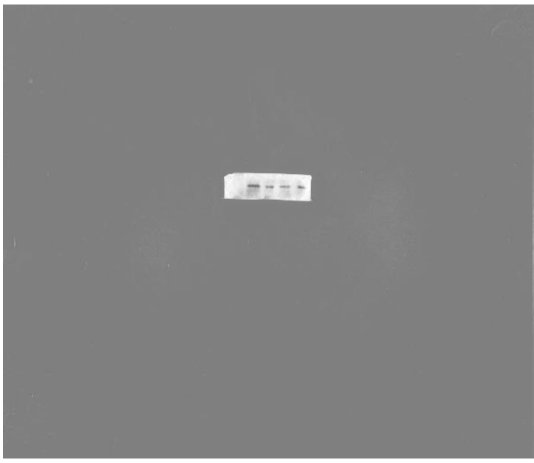

MMP-2

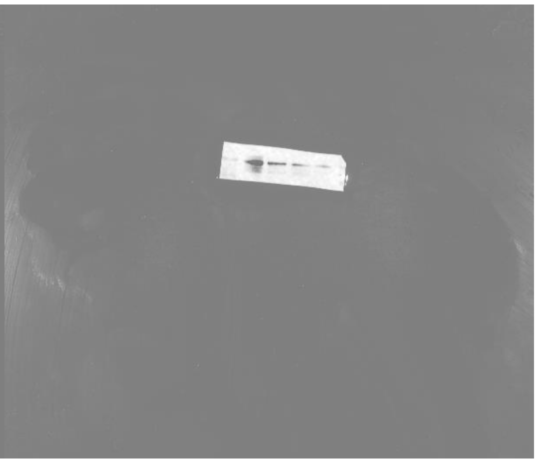

MMP-9

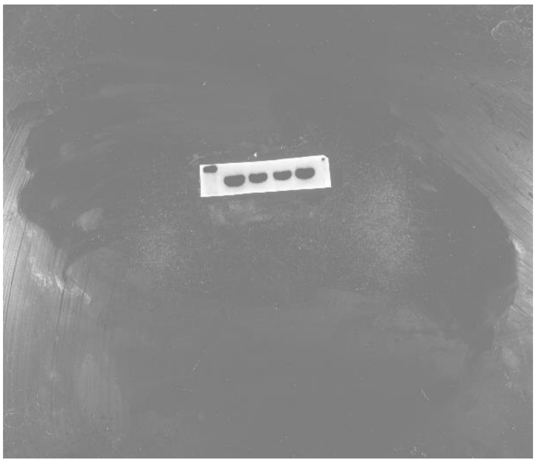

β-Tubulin
